# Comparative oxidative and inflammatory responses to heated tobacco aerosol and cigarette smoke extracts in human lung fibroblasts

**DOI:** 10.3389/fphys.2026.1902675

**Published:** 2026-10-27

**Authors:** Xylia Annisa Abelia, Ronny Lesmana, Melisa Intan Barliana, Massimo Caruso, Riccardo Polosa

**Affiliations:** 1 Doctoral Program in Pharmacy, Faculty of Pharmacy, Universitas Padjadjaran, Bandung, Indonesia; 2 Department of Biological Pharmacy, Faculty of Pharmacy, Universitas Padjadjaran, Bandung, Indonesia; 3 Department of Physiology, Faculty of Medicine, Universitas Padjadjaran, Bandung, Indonesia; 4 Physiology Molecular Laboratory, Biological Activity Division, Central Laboratory, Universitas Padjadjaran, Bandung, Indonesia; 5 Center of Excellence for Pharmaceutical Care Innovation, Universitas Padjadjaran, Bandung, Indonesia; 6 Department of Biomedical and Biotechnological Sciences, University of Catania, Catania, Italy; 7 Center of Excellence for the Acceleration of Harm Reduction (CoEHAR), University of Catania, Catania, Italy; 8 Department of Clinical and Experimental Medicine, University of Catania, Catania, Italy

**Keywords:** *Atg5*, cigarette smoke extract, heated tobacco products, inflammation, lung fibroblasts, oxidative stress, reactive oxygen species

## Abstract

**Introduction:**

Combustible cigarette smoke induces oxidative stress and inflammation in lung cells. Heated tobacco products (HTPs) have been proposed as alternatives with reduced toxicant emissions, but their cellular effects remain incompletely characterized.

**Methods:**

MRC-5 human lung fibroblasts were exposed to aqueous cigarette smoke extract (CSE; 0.1–1.0 puffs/mL) or heated tobacco aerosol extract (HTE; 1.5–5.5 puffs/mL for viability, 3.0–5.5 puffs/mL for other endpoints). Concentrations were anchored to each product’s 24 h IC_50_ (CSE 0.70 puffs/mL, 95% CI 0.63–0.77; HTE 4.76 puffs/mL, 95% CI 4.39–5.16). Cytotoxicity, oxidation-sensitive dihydroethidium (DHE) fluorescence, intracellular TNF-α and IL-6, and morphology were assessed. ATG5 protein was included as an exploratory endpoint.

**Results:**

Both extracts increased DHE fluorescence and intracellular TNF-α and IL-6 in a concentration-dependent manner, with reduced viability and morphological changes. ATG5 findings were inconclusive, as no concentration differed from the untreated control. Nominal puff concentrations were not considered equivalent doses. In an exploratory comparison near each product’s 24 h IC_50_ (CSE 0.7 and HTE 4.75 puffs/mL), CSE produced greater DHE fluorescence (3.17- versus 2.31-fold over vehicle) and greater intracellular IL-6 and TNF-α signals than HTE. Equivalent viability at the 90 min and 4 h endpoints was not established.

**Discussion:**

Under these study-specific conditions, HTE elicited quantitatively smaller oxidative and inflammatory responses than CSE at the selected comparison concentrations.

## 1 Introduction

Cigarette smoke is a major exogenous source of reactive oxygen species (ROS) that disrupt cellular redox balance and induce oxidative stress in lung tissue (Kirkham and Barnes, 2013). Elevated ROS levels are known to contribute to inflammatory signaling and cellular dysfunction, including increased expression of pro-inflammatory cytokines (Cloonan and Choi, 2016). In addition, oxidative stress has been associated with modulation of autophagy-related processes, a conserved cellular mechanism involved in the turnover of damaged organelles and maintenance of cellular homeostasis (Ryter and Choi, 2015). Dysregulation of these stress-response pathways has been implicated in the pathogenesis of chronic lung diseases, including chronic obstructive pulmonary disease (COPD) and pulmonary fibrosis (Chen et al., 2008; Levine and Kroemer, 2019).

Heated tobacco products (HTPs) have been developed as alternative nicotine delivery systems designed to heat, rather than burn, tobacco, thereby reducing the formation of combustion-derived toxicants. Several analytical and toxicological studies have demonstrated that aerosols from HTPs contain markedly lower levels of carbonyl compounds, volatile organic compounds (VOCs), and particulate matter compared with combustible cigarette smoke (Forster et al., 2018; Smith et al., 2021). *In vitro* studies further suggest that HTP emissions may be associated with reduced oxidative stress and inflammatory responses compared to cigarette smoke exposure (Emma et al., 2022). Nevertheless, data on the involvement of autophagy in cellular responses to HTP exposure remain scarce. Since oxidative stress and inflammation are closely linked to multiple cellular stress-response pathways, comparative evaluation of these responses following exposure to HTP aerosols and cigarette smoke may provide further insight into their differential biological effects.

MRC-5 cells, as a widely used human lung fibroblast model, provide a biologically relevant *in vitro* system to investigate cellular stress responses in the respiratory tract. Lung fibroblasts play a central role in oxidative stress responses, inflammatory signaling, and tissue remodeling. Previous studies have highlighted the value of epithelial–fibroblast co-culture systems as relevant models and potential alternatives to animal testing in respiratory toxicology research (Kim et al., 2024). While the oxidative and inflammatory effects of heated tobacco products have been increasingly investigated, the involvement of autophagy-related processes in response to non-combustible nicotine delivery systems remains insufficiently characterized. Therefore, this study compared the oxidative and inflammatory responses induced by cigarette smoke extract (CSE) and heated tobacco aerosol extract (HTE) in MRC-5 cells, with ATG5 protein levels included as an exploratory endpoint. We tested the hypothesis that both products induce concentration-dependent oxidative and inflammatory responses under their respective standard puffing regimens, and that HTE requires higher nominal puff concentrations to do so. Because the regimens differ in puff volume, duration and interval, nominal puff concentrations are not chemically equivalent between products. Two explicit comparison benchmarks were therefore defined in advance: (i) the nominal concentration range of each product under its own standard exposure conditions, and (ii) an exploratory comparison, added during revision, at the concentrations closest to each product’s own 24 h IC_50_.

## 2 Materials and methods

### 2.1 Cell culture

Human lung fibroblast cells (MRC-5, ATCC^®^ CCL-171™, Manassas, VA, USA) were cultured in Dulbecco’s Modified Eagle Medium (DMEM; Thermo Fisher Scientific, Cat. No. 11530556) supplemented with 10% fetal bovine serum (FBS) and 1% penicillin–streptomycin. Cells were maintained at 37 °C in a humidified incubator with 5% CO_2_. For experiments, cells were seeded at a density of 1.7 × 10^5^ cells/mL in either 96-well or 24-well plates and allowed to adhere for 24 h prior to exposure.

### 2.2 Chemical reagents

Reagents used in this study included phosphate-buffered saline (PBS; Sigma-Aldrich, Cat. No. 524650), PrestoBlue™ Cell Viability Reagent (Thermo Fisher Scientific, A13261), dihydroethidium (DHE; Thermo Fisher Scientific, D11347), HEPES buffer (Sigma-Aldrich, H4034), and Antimycin A (Sigma-Aldrich, A8674). For in-cell Western (ICW) analysis, black-walled 96-well plates (LI-COR, D00824-05) and CellTag™ kits (GAM 700, LI-COR 926-42091; GAR 700, LI-COR 926-42092) were used, along with 0.1% Triton™ X-100, 37% formaldehyde, and Tween^®^ 20 (Millipore).

### 2.3 Preparation of cigarette smoke extract (CSE) and heated tobacco aerosol extract (HTE)

Cigarette smoke extract (CSE) was prepared from Marlboro Red cigarettes (Philip Morris International, Indonesia), while heated tobacco aerosol extract (HTE) was generated using an IQOS 3 Duo device and HEETS Sienna heatsticks (Philip Morris International, Indonesia). Commercially available products were selected to reflect real-world exposure conditions.

Smoke and aerosol were generated using standardized smoking and aerosol generation systems (LM1 and LM4E, Körber, Hamburg, Germany). Cigarette smoke was produced following the ISO 3308:2012 regimen (35 mL puff volume, 2 s puff duration, 60 s interpuff interval), whereas IQOS aerosol was generated under the Health Canada Intense (HCI) regimen (55 mL puff volume, 2 s duration, 30 s interpuff interval) in accordance with ISO 20778:2018 (Emma et al., 2022). These regimens were selected to reflect established testing standards for each product category. Generation was carried out in a conditioned environment with an air velocity of 20 cm/s in the vicinity of the product; the LM1 machine operated at 60 ± 3% relative humidity and 22 ± 1 °C, and the LM4E machine at 60 ± 5% relative humidity and 22 ± 2 °C. Due to differences in puffing parameters, direct toxicant yield equivalence cannot be assumed; therefore, the study was designed as a comparative assessment of biological responses under product-appropriate conditions.

Mainstream smoke from 10 cigarettes puffed 8 times each (80 puffs in total) was bubbled directly through 40 mL of ice-cold PBS (pH 7.4), giving a CSE stock of 2 puffs/mL. Mainstream aerosol from 30 heatsticks puffed 12 times each (360 puffs in total) was bubbled directly through 40 mL of ice-cold PBS under identical capture conditions, giving an HTE stock of 9 puffs/mL. Both extracts were generated in the same laboratory (Central Laboratory, Universitas Padjadjaran) with the same PBS as capture liquid, sterile-filtered through a 0.22 µm membrane, and applied to the cells within 30 min of generation; a fresh extract was prepared for each independent biological replicate. Exposure concentrations were obtained by diluting the stock in complete culture medium and are expressed as nominal puff equivalents per mL (puffs/mL); the complete dilution scheme, including the volume of extract and medium used for every concentration, is given in **Supplementary Table S1**. The extracts were not chemically characterized, and pH, osmolality and particulate content were neither measured nor standardized between products; exposure concentrations are therefore reported as nominal values within this experimental framework.

### 2.4 Cell exposure

Working solutions were prepared immediately before exposure by diluting each stock in complete culture medium to a final volume of 1 mL (**Supplementary Table S1**). MRC-5 cells were exposed under standard culture conditions (37 °C, 5% CO_2_). The vehicle control (0 puffs/mL) consisted of 37.5% v/v PBS in culture medium and served as the reference condition for all products and concentrations, except for ATG5, for which the untreated control was retained as the reference (Section 2.9); untreated control wells received culture medium only. Because the puffing regimens and aerosol generation parameters differ between products, doses are nominal exposure metrics rather than directly comparable chemical mass equivalents, and one puff of CSE is not equivalent to one puff of HTE. Exposure durations were 90 min for the DHE assay, 4 h for intracellular protein measurements, 10 h for morphology, and 24 h for viability. For the DHE assay, the 90-min extract exposure was followed by treatment removal and a separate 30-min DHE incubation, as described in Section 2.7.

### 2.5 Cytotoxicity assay

Cell viability was evaluated using the PrestoBlue™ Resazurin Reduction Assay. MRC-5 cells were exposed for 24 h to increasing concentrations of CSE or HTE (CSE: 0.1, 0.3, 0.5, 0.7 and 1.0 puffs/mL; HTE: 1.5, 2.0, 2.5, 3.0, 3.5, 4.0, 4.5, 5.0 and 5.5 puffs/mL). Following exposure, 10 µL of PrestoBlue reagent was added to 90 µL of fresh medium per well and incubated for 2 h at 37 °C. Absorbance was measured at 570 nm and 600 nm using a microplate reader (Lin et al., 2020). Viability was calculated after subtraction of blank absorbance and expressed relative to the vehicle control (0 puffs/mL), which was set to 100%; this assay served as the dose-finding experiment for the concentrations used in the subsequent mechanistic analyses (Balbaied and Moore, 2020).

Half-maximal inhibitory concentrations (IC_50_) were estimated by nonlinear least-squares regression of the biological-replicate viability values on concentration, using a normalized log-logistic model with variable slope in which the top was constrained to 100% (the vehicle) and the bottom to 0%. IC_50_ values are reported with 95% confidence intervals and Hill slopes (**Supplementary Table S2**). An unconstrained four-parameter fit was not used because, for CSE, it returned a non-physical lower asymptote. All assays were performed in three independent biological replicates, each with technical triplicate wells.

### 2.6 Morphological analysis

Cellular morphological alterations were evaluated using phase-contrast microscopy. MRC-5 fibroblasts were seeded in 24-well culture plates and allowed to reach approximately 80% confluence before treatment. Cells were exposed to graded concentrations of CSE (0.1; 0.3; 0.5; 0.7; 1.0 puffs/mL) and HTE (3.0; 4.0; 4.75; 5.0; 5.5 puffs/mL) for 10 h at 37 °C in a humidified atmosphere of 5% CO_2_. The concentrations were the IC_50_-anchored grid used for all mechanistic assays, spanning 0.14–1.43 × IC_50_ for CSE and 0.63–1.16 × IC_50_ for HTE (**Supplementary Table S3**). Morphology was recorded after 10 h, an intermediate time point between the 4 h protein endpoints and the 24 h viability endpoint; because cytotoxicity develops with time, the 24 h IC_50_ was used only as a scaling anchor and not as a prediction of the extent of cell death at 10 h. This interval was chosen because the structural features of interest cell rounding, cytoplasmic condensation and detachment develop later than the biochemical responses, whereas by 24 h the most affected wells had lost enough cells for the remaining monolayer to be unrepresentative; at 10 h these features are already apparent while most cells are still attached and can be imaged in the same field. At the end of exposure, cultures were rinsed with PBS and examined using an inverted microscope (EVOS™ XL Core Imaging System, Thermo Fisher Scientific) at 4× magnification. Morphological features such as cell rounding, shrinkage, and detachment were documented from randomly selected fields. Morphology was assessed qualitatively and is used only as supporting evidence for the quantitative endpoints. Untreated and PBS-treated cells (0 puffs/mL) were used as controls.

### 2.7 Reactive oxygen species (ROS) production

Oxidative stress was examined by measuring intracellular superoxide generation with the dihydroethidium (DHE) probe in MRC-5 fibroblasts following exposure to smoke- and aerosol-derived aqueous extracts, adapted from previously published methods for assessing oxidative stress in human lung cells (Fetterman et al., 2018). Cells were seeded in 96-well plates and allowed to reach approximately 80% confluence over 24 h in DMEM supplemented with 10% fetal bovine serum, 1% penicillin–streptomycin, and 10 mM HEPES specifically for the ROS assay.

Cells were exposed to 200 µL/well of CSE (0.1, 0.3, 0.5, 0.7, and 1.0 puffs/mL) or HTE (3.0, 4.0, 4.75, 5.0, and 5.5 puffs/mL) for 90 min. The treatment solutions were then removed, and cells were incubated with 200 µL/well of DHE (10 µM) for 30 min at 37 °C and 5% CO_2_. Cells were washed three times with 200 µL/well of pre-warmed PBS, followed by the addition of 200 µL/well of fresh PBS. Fluorescence was measured within 30 min using excitation and emission wavelengths of 518 and 606 nm, respectively. Positive-control wells received 200 µL/well of antimycin A (50 µM) for 30 min before treatment removal and DHE staining. Reagent blanks contained medium/buffer and DHE without cells or extract; their fluorescence was approximately 0.4% of the vehicle signal and was subtracted from every reading before analysis (**Supplementary Table S22**). Blank-corrected fluorescence was expressed as fold change relative to the mean of the vehicle wells (0 puffs/mL) within the same biological replicate. DHE staining was performed after extract removal; the 90-min extract exposure and the subsequent 30-min staining period were separate stages. Cell-free extract controls and chromatographic separation of DHE oxidation products were not performed. The 90 min exposure was taken from the published dihydroethidium protocol on which this assay was based (Fetterman et al., 2018) and matches the kinetics of the endpoint: oxidant production by aqueous extracts is rapid, and because the probe was loaded only after the extract had been removed, the signal reflects the intracellular oxidant activity that remained after the preceding exposure rather than a later secondary response. A short exposure also limits cell loss and so reduces the likelihood that fluorescence per well is confounded by a change in cell number, although viability was not measured at this time point. The signal is therefore reported as oxidation-sensitive DHE-associated fluorescence rather than a specific measurement of superoxide.

### 2.8 In-cell western analysis

In-Cell Western (ICW) assay was performed to quantify intracellular protein expression of IL-6, TNF-α, and ATG5. This method was selected to enable quantitative, high-throughput assessment of protein expression while preserving cellular context. Therefore, this approach reflects intracellular protein expression rather than secreted cytokine levels and should be interpreted as an indicator of intracellular inflammatory signaling rather than cytokine secretion. MRC-5 cells were seeded in black-walled, clear-bottom 96-well plates and incubated for 24 h to reach approximately 80% confluence. Cells were then exposed for 4 h to CSE and HTE at concentrations guided by IC_50_-derived dose ranges. Four hours was chosen because it falls within the interval in which intracellular TNF-α and IL-6 protein accumulates after an acute stimulus, while still preceding the extensive detachment seen at longer exposures. Retaining the monolayer is important for this assay because the target signal is normalized to the CellTag 700 cell-content channel measured in the same well, and that normalization becomes unreliable once a large fraction of the cells has been lost; the cell-content values recorded at this time point show that at least 65% of the vehicle cell content was retained at every concentration (**Supplementary Table S17**). Following exposure, cells were immediately fixed by adding 150 µL of 3.7% formaldehyde and incubated for 20 min at room temperature (RT). Wells were washed twice with PBS (pH 7.4) and permeabilized with 0.1% Triton X-100 in PBS for 20 min on a gentle shaker at RT. Non-specific binding was blocked using 150 µL of TBS-based blocking buffer for 30 min at RT. Primary antibody incubation was performed overnight at 4 °C with the following antibodies: Anti-TNF-α (Rabbit, Cell Signaling #3707, 1:1000), Anti-IL-6 (Mouse, Abcam #Ab9324, 1:500), and Anti-ATG5 (Mouse, RnD #MAB5294, 1:500).

Background control wells received antibody diluent only. After primary incubation, plates were washed four times (5 min each) with TBS containing 0.1% Tween-20 (TBS-T) on a shaker at RT. Secondary antibody incubation was then carried out for 1 h at RT in the dark, using IRDye 800CW-conjugated goat anti-rabbit IgG and IRDye 800CW-conjugated goat anti-mouse IgG (LI-COR Biosciences, USA; 1:1000) for detection in the 800 nm channel (green pseudocolor). For normalization, CellTag™ 700 (LI-COR) was used to generate signal in the 700 nm channel (red pseudocolor), representing total cell content. Following secondary incubation, plates were washed four times (5 min each) with TBS-T in the dark and scanned using an Odyssey CLx Infrared Imaging System (LI-COR Biosciences, USA). Imaging was performed at 169 µm resolution, with intensity settings of 5.0 (700-channel) and 7.0 (800-channel), and an offset of 4.0 mm. Fluorescent signals were quantified using Odyssey Imaging Software v3.0, and average 800-channel intensities were normalized to the 700-channel signals to correct for well-to-well variation in cell number. Both the background-corrected 800 nm signal and the 800/700 ratio are reported, because a normalized ratio can rise when the 700 nm signal falls as a result of cell loss (**Supplementary Table S17**).

### 2.9 Statistical analysis

Data are expressed as mean ± standard deviation (SD) of three independent biological replicates. Each condition was measured in technical triplicate wells within every biological replicate; technical replicates were averaged within each replicate, and all statistical tests were performed on the resulting biological-replicate means (n = 3 per group). For the in-cell Western endpoints, CSE and HTE were exposed on the same plate and shared the untreated-control and vehicle wells; these endpoints were therefore analyzed as a single family comprising the vehicle and all ten treatment concentrations. The dihydroethidium and PrestoBlue assays were run on separate plates for each product and were analyzed per product. Group differences were assessed by one-way ANOVA, with normality of residuals checked by the Shapiro–Wilk test and homogeneity of variance by Levene’s test (median-centered); neither assumption was rejected for any endpoint (**Supplementary Table S6**). Each concentration was then compared with the vehicle using Dunnett’s two-sided many-to-one test, and the vehicle was compared with the untreated control by Welch’s t-test. For ATG5 the untreated control was retained as the reference condition; each concentration was compared with it using the same Dunnett procedure, and the vehicle-referenced comparisons are reported alongside them because the vehicle wells of that plate carried an anomalous signal (**Supplementary Table S13**; **Supplementary Section S4**). The background-corrected 800 nm channel and the 700 nm cell-content channel of the in-cell Western endpoints were analyzed in the same way (**Supplementary Table S17**). For the dihydroethidium assay, inference was performed on blank-corrected fluorescence values, because normalizing to the vehicle within each biological replicate fixes the vehicle at one and removes its variance; the figures display fold changes. Effect sizes are reported as η² and ω², and concentration–response trends were assessed by linear regression on log_10_ concentration with Spearman’s rank correlation as a distribution-free check. Whether a single relative-potency factor could describe the difference between products was tested by parallel-line analysis (ANCOVA on log_10_ concentration with a product × concentration interaction); because parallelism was rejected, the products were instead compared, in an exploratory analysis, at the tested concentrations closest to each product’s own 24 h IC_50_ (CSE 0.7 and HTE 4.75 puffs/mL), by paired t-test for the in-cell Western endpoints and Welch’s t-test for dihydroethidium. Analyses were performed in Python 3.11 with SciPy 1.17. Exact p-values are reported, except that Dunnett-adjusted p-values below 0.001 are given as p < 0.001, because they are obtained by numerical integration and are not stable beyond that level; p < 0.05 was considered statistically significant. Full statistical output is provided in **Supplementary Tables S4**–**S16**.

## 3 Results

### 3.1 Comparative analysis of cytotoxicity in MRC-5 cells following exposure to CSE and HTE

Exposure of MRC-5 cells to CSE and HTE produced a concentration-dependent reduction in cell viability (**Figure 1**). Relative to the vehicle control, CSE reduced viability significantly from 0.5 puffs/mL onwards (68.6 ± 15.3%, p = 0.004; 57.3 ± 5.1% at 0.7 puffs/mL, p < 0.001; 20.4 ± 4.4% at 1.0 puffs/mL, p < 0.001), whereas HTE became significant from 3.5 puffs/mL onwards (65.8 ± 11.6%, p = 0.005) and reached 38.2 ± 7.5% at 5.5 puffs/mL (p < 0.001); HTE at 2.5 puffs/mL or below did not differ from the vehicle (Dunnett test; **Supplementary Tables S7**, **S8**). Nonlinear regression of the dose–response curves gave an IC_50_ of 0.70 puffs/mL (95% CI 0.63–0.77; Hill slope 2.90) for CSE and 4.76 puffs/mL (95% CI 4.39–5.16; Hill slope 3.48) for HTE (**Supplementary Table S2**). Under the present exposure conditions, HTE therefore required higher nominal puff concentrations than CSE to reduce viability to a comparable extent. Because the two products were generated under different puffing regimens, this difference is expressed in nominal puff units and does not represent a difference per unit of delivered toxicant mass.

**Figure 1:**
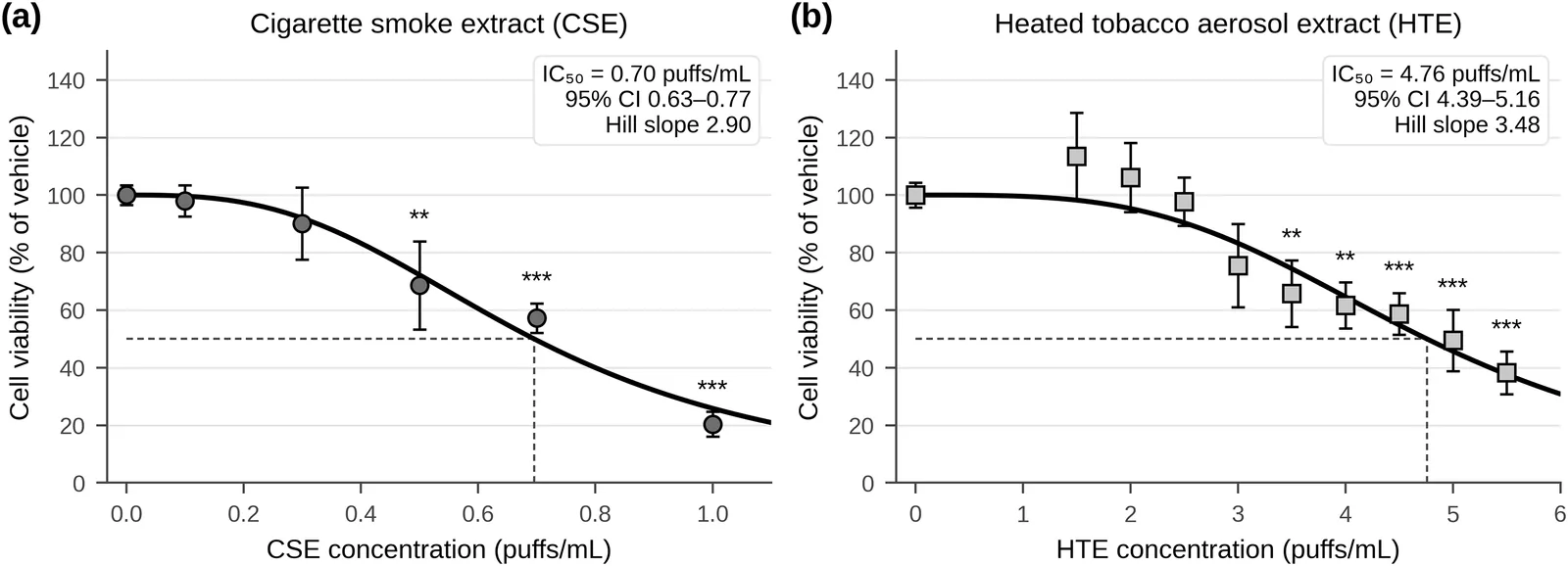
Dose–response curves of cigarette smoke extract (CSE) and heated tobacco aerosol extract (HTE) in MRC-5 cells. Dose–response curve of **(a)** CSE; **(b)** HTE following 24 h exposure. Cell viability was assessed using the PrestoBlue™ assay and expressed as percentage of the vehicle control (0 puffs/mL, 37.5% v/v PBS). Both extracts reduced viability in a concentration-dependent manner. Curves are normalized log-logistic fits with variable slope, giving IC_50_ values of 0.70 puffs/mL (95% CI 0.63–0.77) for CSE and 4.76 puffs/mL (95% CI 4.39–5.16) for HTE. Data are means ± SD of three independent biological replicates. Asterisks indicate differences from the vehicle control (Dunnett test): **p < 0.01, ***p < 0.001.

### 3.2 Morphological analysis following CSE and HTE exposure

Exposure to CSE and HTE resulted in morphological alterations in MRC-5 cells following exposure to CSE and HTE (**Figure 2**). Control cells exhibited the characteristic spindle-shaped morphology with intact intercellular junctions and a confluent monolayer. Exposure to increasing concentrations of CSE (**Figure 2a**) resulted in progressive structural changes, including cell shrinkage, rounding, and detachment. At higher concentrations, a greater number of rounded and condensed cells were observed, along with increased cellular debris.

**Figure 2:**
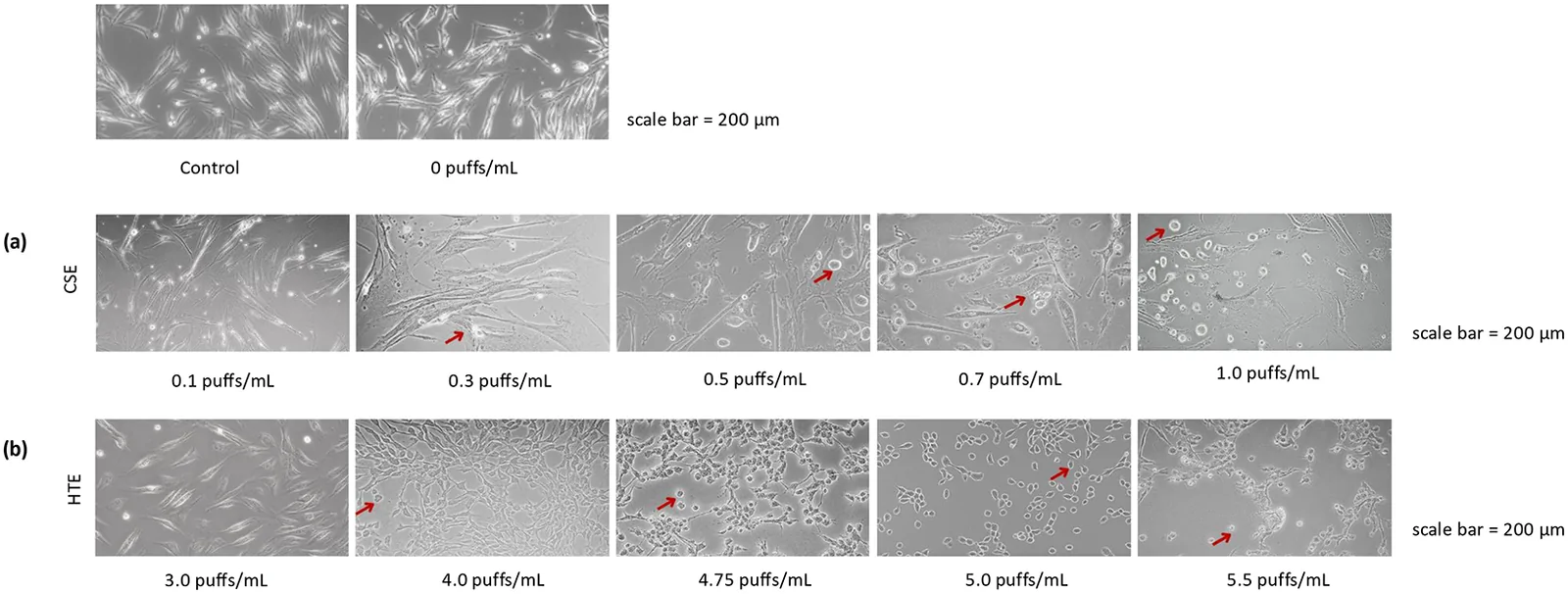
Representative phase-contrast micrographs of MRC-5 cells exposed to **(a)** cigarette smoke extract (CSE) and **(b)** heated tobacco aerosol extract (HTE). Control cells exhibit the typical elongated fibroblast morphology with intact cell–cell contacts. CSE exposure resulted in concentration-dependent morphological alterations, including cell shrinkage, rounding, and detachment, with increased cellular debris at higher concentrations. HTE exposure induced more gradual morphological changes, with cells largely maintaining attachment at lower concentrations and exhibiting increased rounding and debris at higher concentrations. Red arrows indicate representative examples of rounded and condensed cells. Images acquired at 4× objective using an EVOS imaging system; scale bar = 200 µm.

In contrast, HTE-treated cells (**Figure 2b**) displayed a more gradual morphological response. At lower concentrations, cells largely retained their normal morphology and attachment. With increasing concentrations, features such as cytoplasmic condensation and cell rounding became evident, while a substantial proportion of cells remained adherent. At higher exposures, an increase in rounded cells and cellular debris was observed. Overall, morphological changes occurred progressively with increasing concentrations for both CSE and HTE, with more pronounced alterations observed in CSE-treated cells at lower nominal concentrations.

### 3.3 Assessment of ROS production induced by CSE and HTE

Oxidation-sensitive DHE fluorescence, used here as an index of intracellular oxidant (ROS) activity, increased with concentration for both products (**Figure 3**). CSE raised the DHE signal from 1.47-fold over vehicle at 0.1 puffs/mL to 3.52-fold at 1.0 puffs/mL, and HTE from 1.77-fold at 3.0 puffs/mL to 2.73-fold at 5.5 puffs/mL. Every concentration of both products differed from the vehicle (Dunnett test on blank-corrected fluorescence, all p < 0.001; **Supplementary Tables S9**, **S10**), and both series showed a significant concentration trend (p ≤ 7.6 × 10^-8^; **Supplementary Table S14**). Antimycin A, the positive control, produced the largest signal (4.51-fold for the CSE plate and 4.57-fold for the HTE plate), confirming assay responsiveness. Across the nominal concentration ranges tested, HTE required higher puff concentrations than CSE to reach comparable DHE signals.

**Figure 3:**
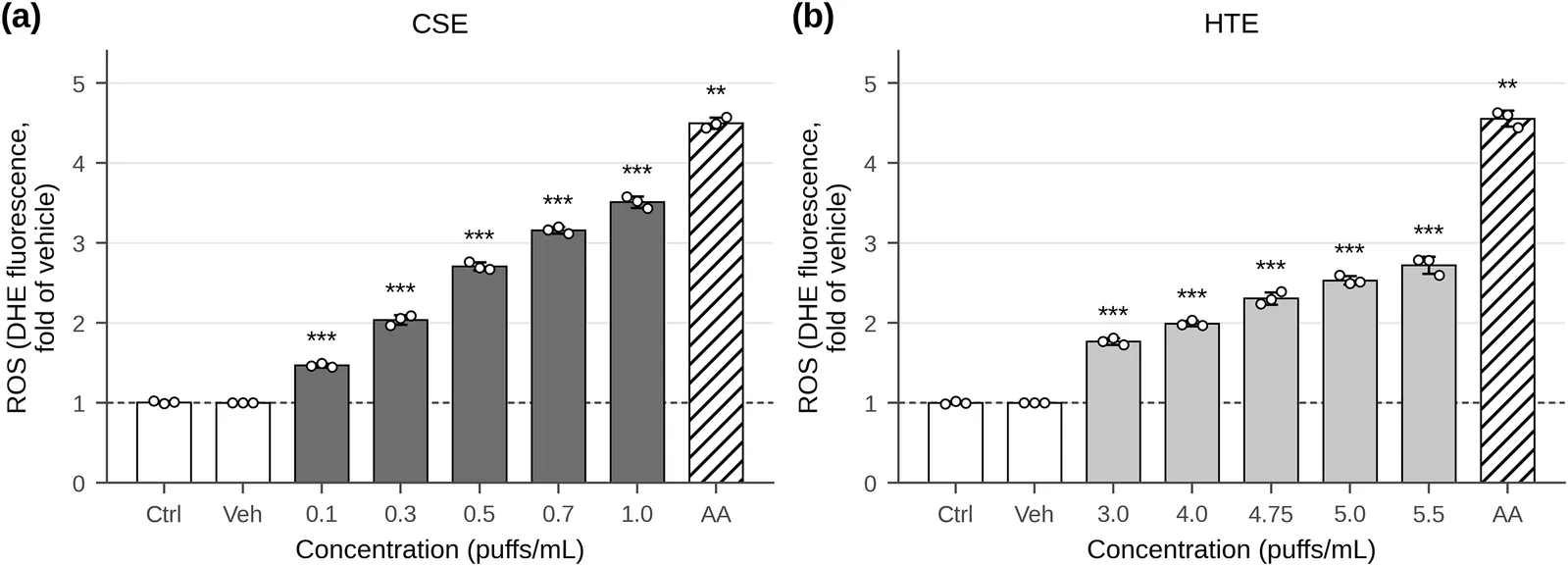
Concentration-dependent intracellular ROS production in MRC-5 cells following exposure to **(a)** cigarette smoke extract (CSE) and **(b)** heated tobacco aerosol extract (HTE). Cells were exposed to increasing concentrations of CSE (0.1–1.0 puffs/mL) and HTE (3.0–5.5 puffs/mL). DHE-associated fluorescence was measured and expressed as fold change relative to the vehicle control (0 puffs/mL, 37.5% v/v PBS). Antimycin A (AA) was used as a positive control. Data are presented as mean ± SD from three independent biological replicates. Statistical analysis was performed by one-way ANOVA on blank-corrected fluorescence values followed by Dunnett’s test against the vehicle control. Asterisks indicate significant differences from the vehicle (**p < 0.01, ***p < 0.001; ** refers to antimycin A); Antimycin A was compared with the vehicle by Welch’s t-test. Open circles show the individual biological-replicate values; full output is given in **Supplementary Tables S9**, **S10**.

### 3.4 Inflammatory cytokine response to CSE and HTE

Quantitative ICW analysis showed a concentration-dependent increase in intracellular TNF-α in MRC-5 cells exposed to both products (representative images in **Figure 4**; quantification in **Figure 5**). Normalized TNF-α increased from 1.30-fold over vehicle at CSE 0.1 puffs/mL to 2.63-fold at 1.0 puffs/mL, and from 1.16-fold at HTE 3.0 puffs/mL to 2.34-fold at 5.5 puffs/mL; all concentrations of both products differed from the shared vehicle control (Dunnett test, all p < 0.001; **Supplementary Table S11**). The same direction of change was present in the background-corrected 800 nm signal before normalization (up to 1.83-fold for CSE and 1.91-fold for HTE), indicating that the increase is not an artefact of normalization; because cell content fell to 0.70–0.81 of the vehicle at the highest concentrations, the normalized ratio exceeds the absolute fold change at those concentrations (**Supplementary Table S17**).

**Figure 4:**
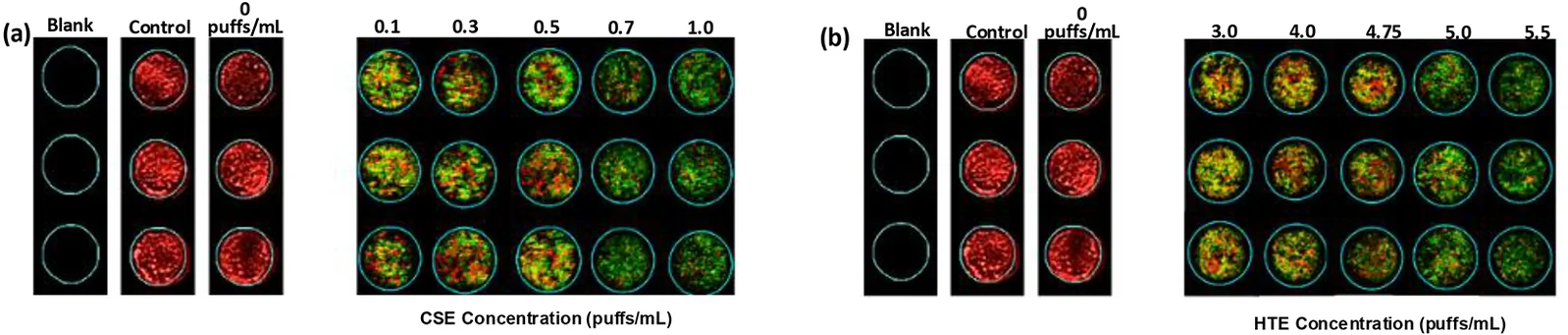
Representative in-cell Western (ICW) fluorescence images of intracellullar TNF-α in MRC-5 cells following exposure to **(a)** cigarette smoke extract (CSE) and **(b)** heated tobacco aerosol extract (HTE). MRC-5 cells were exposed to increasing concentrations of CSE (0.1–1.0 puffs/mL) and HTE (3.0–5.5 puffs/mL). Intracellular TNF-α is shown in the 800 nm channel, with total cell content visualized in the 700 nm channel. Overlay images represent merged fluorescence signals. Because CSE and HTE were exposed on the same plate, the blank, untreated-control and vehicle (0 puffs/mL) wells are shared and are therefore shown in both panels.

**Figure 5:**
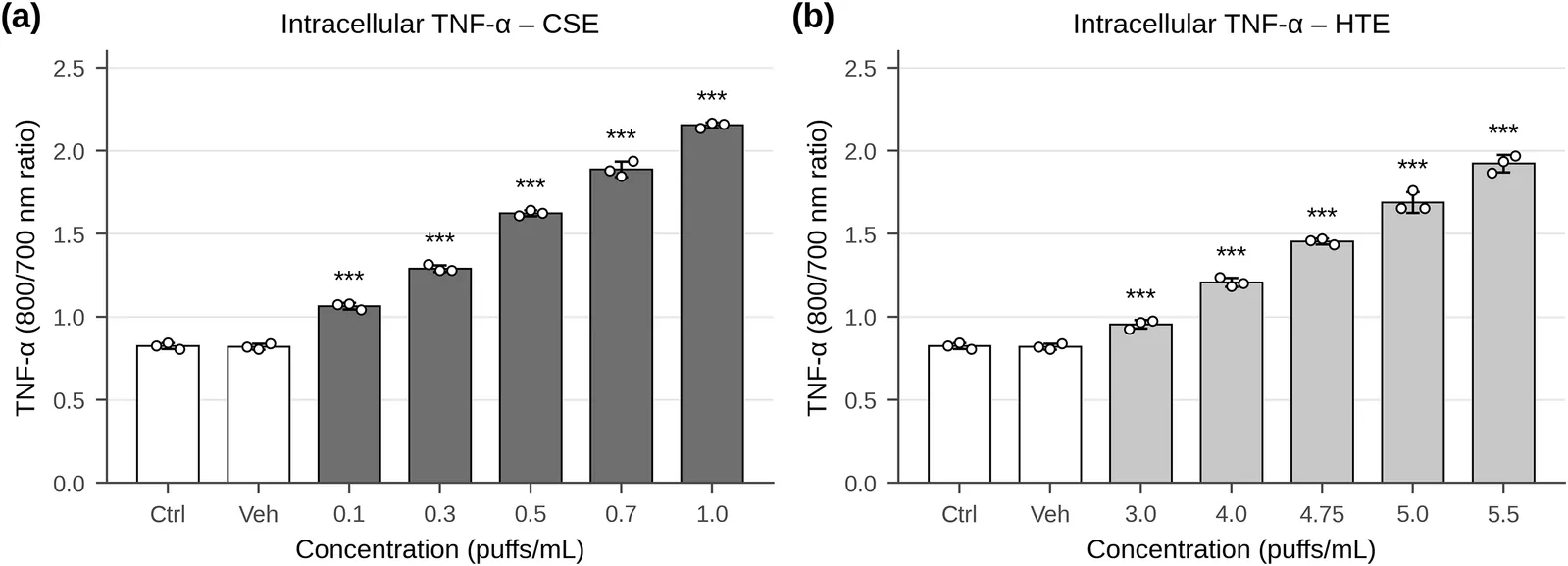
Relative TNF-α expression in MRC-5 cells following exposure to **(a)** cigarette smoke ex-tract (CSE) and **(b)** heated tobacco aerosol extract (HTE). Quantitative analysis of intracellular TNF-α measured by in-cell Western (ICW) assay in MRC-5 cells exposed to increasing concentrations of CSE (0.1–1.0 puffs/mL) and HTE (3.0–5.5 puffs/mL). Fluorescence signals were normalized to total cell content (700 nm channel) and expressed as relative expression. Data are presented as mean ± SD from three independent biological replicates. CSE and HTE were exposed on the same plate and shared the control and vehicle wells; statistical analysis was performed by one-way ANOVA followed by Dunnett’s test against the shared vehicle control. Asterisks indicate significant differences from the vehicle (***p < 0.001). Open circles show the individual biological-replicate values; full output is given in **Supplementary Table S11**.

Intracellular IL-6 followed a comparable pattern (representative images in **Figure 6**; quantification in **Figure 7**), rising from 1.32-fold over vehicle at CSE 0.1 puffs/mL (p = 0.026) to 3.25-fold at 1.0 puffs/mL, and from 1.04-fold at HTE 3.0 puffs/mL (not significant) to 2.95-fold at 5.5 puffs/mL. For HTE, IL-6 became significant from 4.0 puffs/mL onwards (p = 0.007; **Supplementary Table S12**). Increases were again present in the absolute 800 nm signal (up to 2.45-fold for CSE and 2.27-fold for HTE). For both cytokines, the responses of CSE occurred at lower nominal concentrations than those of HTE, but the two concentration scales are not chemically equivalent, so this comparison is restricted to nominal puff units under product-specific exposure conditions.

**Figure 6:**
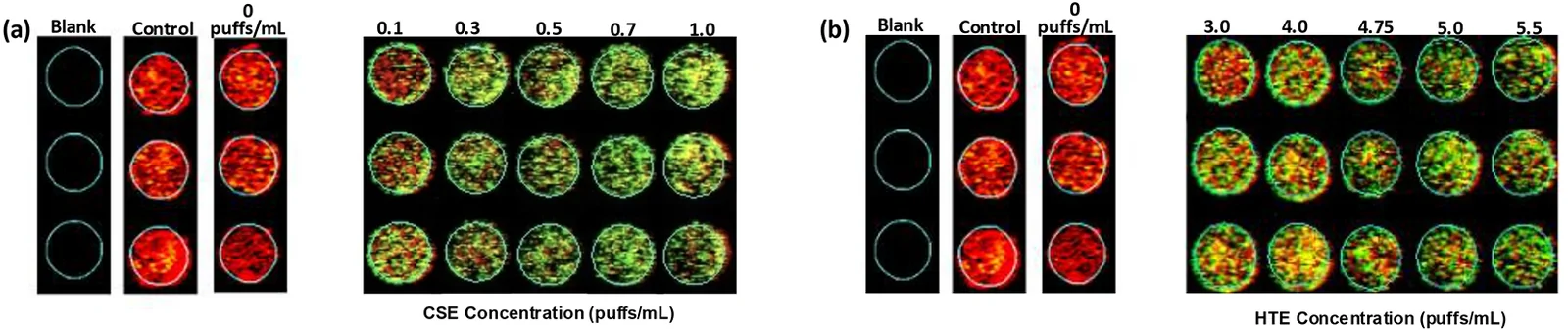
Representative in-cell Western (ICW) fluorescence images of IL-6 expression in MRC-5 cells following exposure to **(a)** cigarette smoke extract (CSE) and **(b)** heated tobacco aerosol extract (HTE). MRC-5 cells were exposed to increasing concentrations of CSE (0.1–1.0 puffs/mL) and HTE (3.0–5.5 puffs/mL). Intracellular IL-6 is shown in the 800 nm channel, with total cell content visualized in the 700 nm channel. Overlay images represent merged fluorescence signals. Because CSE and HTE were exposed on the same plate, the blank, untreated-control and vehicle (0 puffs/mL) wells are shared and are therefore shown in both panels.

**Figure 7:**
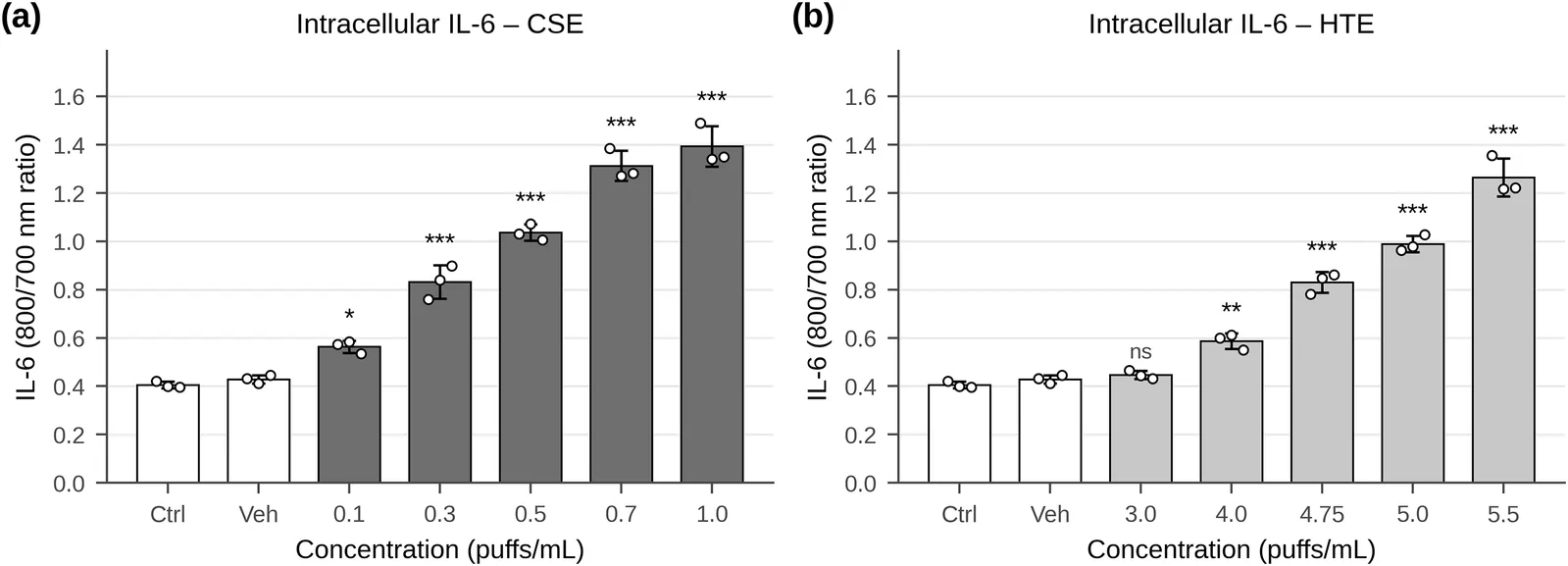
Relative IL-6 expression in MRC-5 cells following exposure to **(a)** cigarette smoke extract (CSE) and **(b)** heated tobacco aerosol extract (HTE). Quantitative analysis of IL-6 expression measured by in-cell Western (ICW) assay in MRC-5 cells exposed to increasing concentrations of CSE (0.1–1.0 puffs/mL) and HTE (3.0–5.5 puffs/mL). Fluorescence signals were normalized to total cell content (700 nm channel) and expressed as relative expression. Data are presented as mean ± SD from three independent biological replicates. CSE and HTE were exposed on the same plate and shared the control and vehicle wells; statistical analysis was performed by one-way ANOVA followed by Dunnett’s test against the shared vehicle control (*p < 0.05, **p < 0.01, ***p < 0.001; ns, not significant). Open circles show the individual biological-replicate values; full output is given in **Supplementary Table S12**.

### 3.5 ATG5 protein expression following CSE and HTE exposure

ATG5 protein levels were measured as an exploratory endpoint (representative images in **Figure 8**; quantification in **Figure 9**). No concentration of either product differed from the untreated control (Dunnett test, all p ≥ 0.35 for CSE and p ≥ 0.95 for HTE; **Supplementary Table S13**). The vehicle wells of this plate carried an ATG5 signal only 36% of that of the untreated control (p < 0.001), even though their cell content was equivalent, whereas vehicle and untreated control did not differ for TNF-α or IL-6 on the same plate layout; a technical problem affecting those wells is the more likely explanation, but its cause could not be established retrospectively and a target-specific effect of the vehicle cannot be excluded (**Supplementary Section S4**). Relative to the untreated control, the absolute ATG5 signal did not increase at any concentration, and the apparent rise in the normalized ratio tracked the fall in cell content. Relative to the untreated control, these data therefore provide no evidence of an increase in ATG5 protein; the apparent rise against the vehicle reflects the anomalous vehicle wells and the falling cell-content denominator rather than a change in ATG5 itself. ATG5 is reported as an exploratory endpoint, and no inference about autophagic activity or flux can be drawn from a single marker.

**Figure 8:**
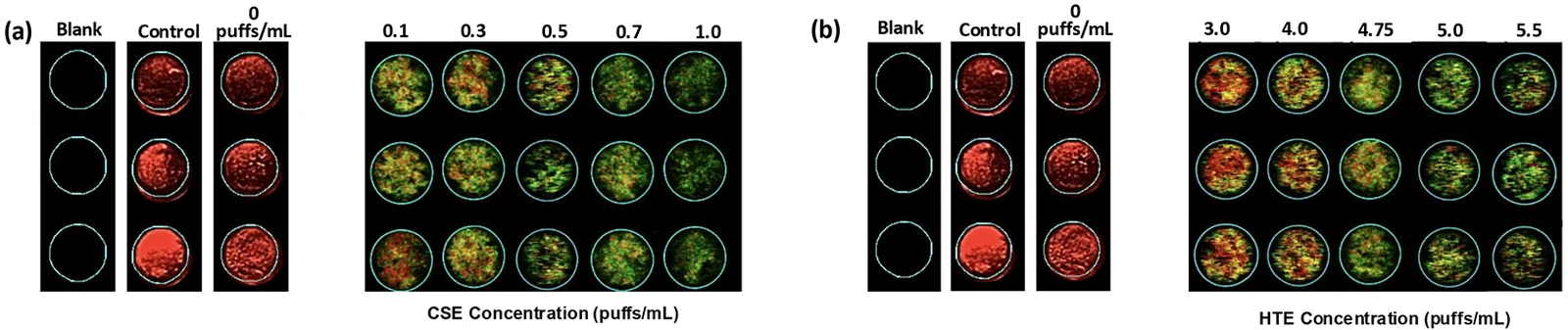
Representative in-cell Western (ICW) fluorescence images of ATG5 expression in MRC-5 cells following exposure to **(a)** cigarette smoke extract (CSE) and **(b)** heated tobacco aerosol extract (HTE). MRC-5 cells were exposed to increasing concentrations of CSE (0.1–1.0 puffs/mL) and HTE (3.0–5.5 puffs/mL). Intracellular ATG5 is shown in the 800 nm channel, with total cell content visualized in the 700 nm channel. Overlay images represent merged fluorescence signals. Because CSE and HTE were exposed on the same plate, the blank, untreated-control and vehicle (0 puffs/mL) wells are shared and are therefore shown in both panels.

**Figure 9:**
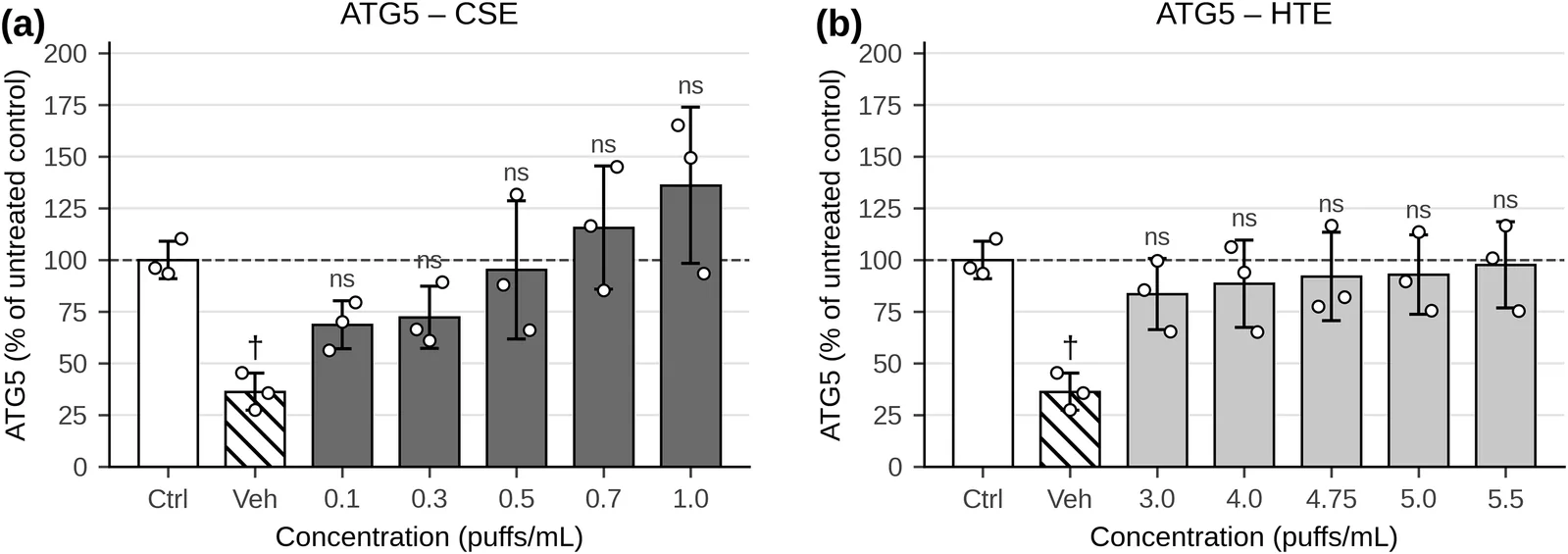
Relative ATG5 protein expression in MRC-5 cells following exposure to **(a)** cigarette smoke extract (CSE) and **(b)** heated tobacco aerosol extract (HTE). Quantitative analysis of ATG5 expression measured by in-cell Western (ICW) assay in MRC-5 cells exposed to increasing concentrations of CSE (0.1–1.0 puffs/mL) and HTE (3.0–5.5 puffs/mL). Fluorescence signals were normalized to total cell content (700 nm channel) and are expressed as a percentage of the mean untreated-control value across the three biological replicates, the same normalization as in **Supplementary Table S18**. Data are presented as mean ± SD from three independent biological replicates. No concentration of either product differed from the untreated control (one-way ANOVA followed by Dunnett’s test; **Supplementary Table S13**); ns, not significant versus the untreated control. Open circles show the individual biological-replicate values. The vehicle wells of this plate (†, hatched bar) carried an anomalously low signal at equivalent cell content and are therefore shown for completeness but not used as the reference (**Supplementary Section S4**).

### 3.6 Exploratory comparison of the two products near each product’s 24 h IC_50_

Because the nominal puff concentrations of the two products are not chemically equivalent, and because the concentration–response lines of CSE and HTE were not parallel on a log_10_ concentration scale (parallelism rejected for ROS, TNF-α, IL-6 and viability, all p ≤ 0.002; **Supplementary Table S15**), no single relative-potency factor can describe the difference between them. The products were therefore also compared, in an exploratory analysis, at the tested concentrations closest to each product’s own 24 h IC_50_ (CSE 0.7 and HTE 4.75 puffs/mL). Their similar cytotoxicity rests on the fitted 24 h IC_50_ values and was not tested at the 90 min DHE or 4 h ICW time points. At these concentrations (**Figure 10**), CSE produced a larger DHE response than HTE (3.17 ± 0.04 versus 2.31 ± 0.08-fold over vehicle, p = 3.6 × 10^-4^), a larger IL-6 signal (3.06 ± 0.17 versus 1.94 ± 0.12-fold, p = 0.015; absolute signal 2.36 versus 1.70-fold, p = 0.023) and a larger normalized TNF-α signal (2.30 ± 0.01 versus 1.77 ± 0.06-fold, p = 0.005), although for TNF-α the difference in the absolute signal did not reach significance (1.80 versus 1.55-fold, p = 0.087). ATG5 did not differ between products (**Supplementary Table S16**). At these concentrations the HTE wells contained a larger extract fraction, and therefore less culture medium, than the CSE wells (52.8% versus 35% v/v), and the single 37.5% PBS vehicle matched neither; this unmatched medium dilution is a limitation of the comparison (Section 4.1). This comparison remains conditional on the exposure conditions of this study and does not establish equivalence of chemical dose.

**Figure 10:**
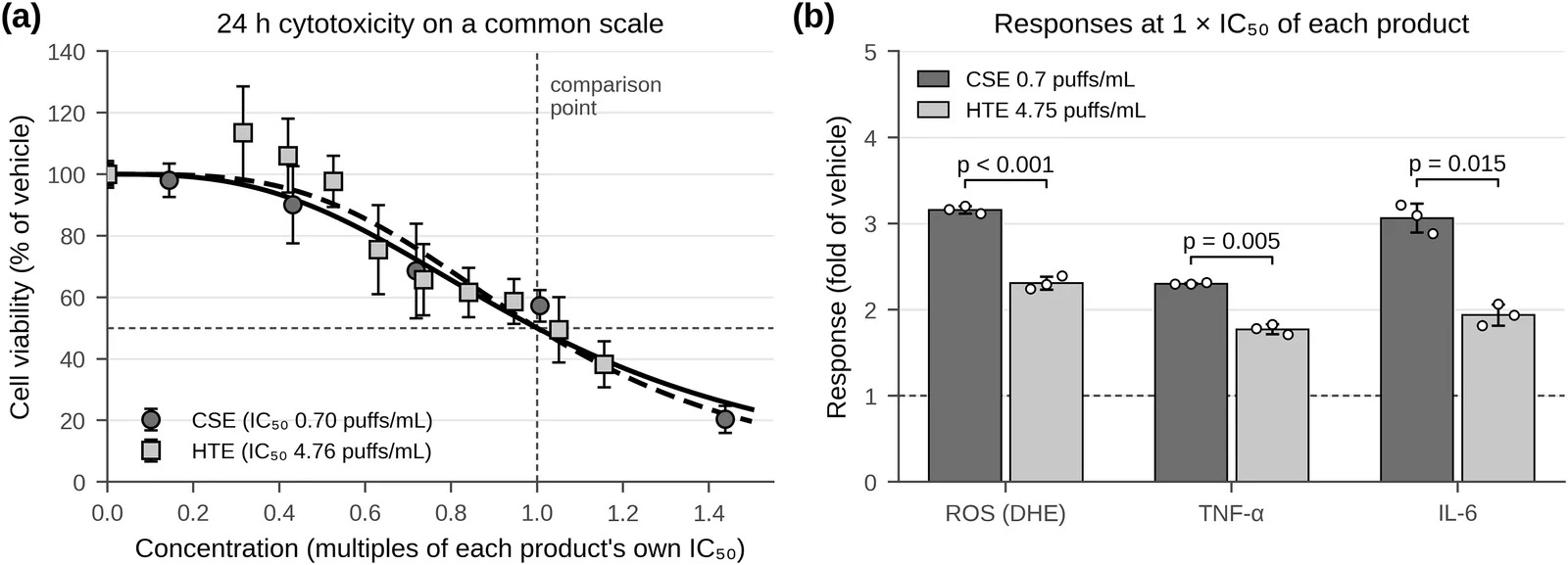
Exploratory comparison of cigarette smoke extract (CSE) and heated tobacco aerosol extract (HTE) near each product’s 24 h IC_50_. **(a)** Cell viability after 24 h plotted against concentration expressed as multiples of each product’s own IC_50_ (CSE 0.70 and HTE 4.76 puffs/mL; **Supplementary Table S2**), which places the two products on a common scale of 24 h cytotoxicity; curves are the normalized log-logistic fits (solid line, CSE; long-dashed line, HTE) and the thin dashed lines mark the comparison point at 1 × IC_50_. **(b)** Oxidation-sensitive dihydroethidium fluorescence (ROS index), intracellular TNF-α and IL-6 measured at the concentrations closest to 1 × IC_50_ of each product (CSE 0.7 and HTE 4.75 puffs/mL), expressed as fold change relative to the vehicle control. Data are means ± SD of three independent biological replicates, with the individual replicate values shown as open circles. p values are from paired t-tests for the in-cell Western endpoints, which were exposed on the same plate within each biological replicate, and from Welch’s t-test for dihydroethidium, which was run on separate plates (**Supplementary Table S16**). Equal cytotoxicity at the 90 min and 4 h assay times was not tested. ATG5 is not shown: it did not differ from the untreated control at any concentration, and the vehicle reference on that plate was unreliable (**Supplementary Section S4**).

## 4 Discussion

The present study demonstrates that exposure to cigarette smoke extract (CSE) and heated tobacco aerosol extract (HTE) induces concentration-dependent alterations in oxidative and inflammatory responses in MRC-5 fibroblasts, while ATG5 protein showed no evidence of a change. Across the endpoints that responded, CSE elicited responses at lower nominal puff concentrations, and in an exploratory comparison at the concentrations closest to each product’s own 24 h IC_50_ it produced the larger responses. Because each product was generated under its own standard puffing regimen and the extracts were not chemically characterized, these comparisons describe biological responses under product-specific exposure conditions rather than responses per unit of delivered toxicant.

Oxidative stress represents a central mechanism underlying cellular responses to tobacco-derived products. Cigarette smoke has been widely reported to generate substantial amounts of reactive oxygen species (ROS), leading to mitochondrial dysfunction, lipid peroxidation, and disruption of cellular redox balance (Fetterman et al., 2018; Demirbağ-Sarıkaya et al., 2021). In line with these observations, the present study demonstrates a pronounced increase in intracellular ROS following CSE exposure. Although HTE exposure resulted in comparatively lower ROS levels, measurable oxidative responses were still observed, suggesting that reduced emissions do not equate to an absence of biological activity. These findings are consistent with previous studies reporting attenuated, yet persistent, oxidative effects associated with heated tobacco aerosols (Jeffery et al., 2018; Ibañez et al., 2019).

The differential oxidative response was paralleled by changes in inflammatory signaling. Both CSE and HTE increased the intracellular levels of the pro-inflammatory cytokines TNF-α and IL-6 in a concentration-dependent manner, with responses to CSE occurring at lower nominal puff concentrations and being larger at the concentrations closest to each product’s 24 h IC_50_. This is in line with established evidence linking oxidative stress to inflammatory activation in lung cells (Carnevali et al., 2003; Kirkham and Barnes, 2013), and further supported by recent studies demonstrating enhanced inflammatory responses following cigarette smoke compared with heated tobacco aerosol exposure (Distefano et al., 2024).

ATG5 protein levels were included as an exploratory endpoint, and the present data provide no evidence that they changed. The answer depends on the reference condition: no concentration differed from the untreated control, whereas several concentrations differed from the vehicle. Across CSE concentrations the signal showed a significant positive trend, tested on the untreated-control scale (**Supplementary Table S14**), so the disagreement between the two references concerns the comparisons at individual concentrations rather than the overall trend. The two references disagree because the vehicle wells of that plate carried an ATG5 signal only about a third of the untreated control at equivalent cell content, while behaving normally for TNF-α and IL-6; the reason could not be identified retrospectively, and although a technical cause is the more likely explanation, it cannot be separated from a target-specific effect of the vehicle (**Supplementary Section S4**). The progressive fall in the cell-content signal that forms the denominator of the normalized ratio is a further reason for caution, since it raises the ratio independently of the target. We therefore report no evidence of an increase in ATG5 protein under these conditions, while noting that a single endpoint of this kind cannot establish the absence of a change, and draw no conclusion about autophagy in this model. That caution would apply even to a clean result, because the relationship between oxidative stress and autophagy is context-dependent (Redza-Dutordoir and Averill-Bates, 2021) and ATG5 also has functions outside macroautophagy, including in the cellular response to DNA damage (Demirbağ-Sarıkaya et al., 2021, 2022). Assessment of autophagy requires flux-based measurements, such as the LC3B-II/LC3B-I ratio and p62 turnover with and without lysosomal inhibition, which were outside the scope of this study; single markers such as ATG5 do not reflect autophagic flux or its functional consequences (Klionsky et al., 2021).

The differential effects observed between CSE and HTE can be partially explained by their distinct physicochemical characteristics. Conventional cigarettes rely on combustion processes reaching temperatures above 800 °C, generating thousands of toxic compounds, whereas heated tobacco products operate at lower temperatures (<350 °C) and produce aerosols through thermal decomposition (Bekki et al., 2017; Ibañez et al., 2019). This results in a substantial reduction in the number and concentration of harmful constituents. Nevertheless, several biologically active compounds, including nicotine and toxic carbonyl species, remain present in HTP emissions (Distefano et al., 2024). Consequently, while HTPs are often considered reduced-risk alternatives, they cannot be regarded as risk-free. Because the two emissions differ qualitatively as well as quantitatively — combustion-derived tar, polycyclic aromatic hydrocarbons, aldehydes and metals in cigarette smoke, against nicotine, glycerol and propylene glycol with lower levels of aldehydes and nitrosamines in heated tobacco aerosol — the responses to the two products are discussed separately here, and the fact that both raised ROS and cytokine levels is not taken as evidence of a shared mechanism. The present design measured a small set of endpoints in parallel and cannot establish a causal sequence linking oxidative stress to inflammatory signaling in either product.

Importantly, variability across studies investigating HTP toxicity has been reported, likely reflecting differences in aerosol generation protocols, exposure systems, and analytical approaches (Jeffery et al., 2018; Demirbağ-Sarıkaya et al., 2021). Such methodological variability may contribute to discrepancies in reported toxicity profiles and highlights the need for standardized experimental frameworks when comparing conventional and alternative tobacco products. Despite these differences, a consistent trend emerges in which HTPs exhibit reduced but detectable biological effects compared to conventional cigarette smoke.

Taken together, both cigarette smoke and heated tobacco aerosol extracts induced measurable oxidative and inflammatory responses in MRC-5 fibroblasts, with larger responses to CSE at the concentrations compared, whereas ATG5 protein showed no evidence of a change. This pattern is consistent with previous reports that combustion-derived products exert greater biological effects, while aerosol-based exposures are reduced in toxicity but not biologically inert (Hashizume et al., 2023; Distefano et al., 2024). The use of puff-based exposure metrics is a central limitation, as chemical equivalence between products cannot be established; physicochemical characterization of the extracts, including nicotine and carbonyl measurements, was not performed. The findings should therefore be read as a comparison of biological responses under product-specific exposure conditions. Studies incorporating chemically anchored dose metrics, multiple molecular endpoints, functional assays and more physiologically relevant exposure models are needed to clarify the underlying pathways and their longer-term implications.

### 4.1 Limitations

Interpretation is limited by the absence of chemical characterization, non-standardized pH and osmolality, different puffing regimens, and unmatched medium/serum dilution with the fixed PBS vehicle. Nominal puff concentrations therefore do not establish dose equivalence. MRC-5 cells are a single fetal lung fibroblast line and do not recapitulate adult lung physiology, epithelial–immune crosstalk, or the microenvironment of COPD and pulmonary fibrosis; the acute, submerged exposure to aqueous extracts also differs from repeated whole-aerosol exposure at the air–liquid interface, and more physiologically representative exposure models were not used, so chronic, multicellular lung responses are not captured. DHE fluorescence is not superoxide-specific, and extract interference was not assessed. Intracellular cytokine measurements do not establish secretion and lacked independent validation. The ATG5 comparison depended on the reference condition and the vehicle wells of that plate were anomalous, so the absence of an increase is established only against the untreated control; a single marker cannot establish autophagic flux. Comparisons anchored to the 24 h IC_50_ remain exploratory because equivalent cytotoxicity at earlier endpoints was not established; viability curves were not obtained at the 90 min, 4 h and 10 h assay times. Three biological replicates limit precision, and these findings do not establish relative product safety or population-level risk.

## 5 Conclusion

In summary, the present findings demonstrate that both cigarette smoke extract (CSE) and heated tobacco aerosol extract (HTE) induce measurable cellular responses in human lung fibroblasts, characterized by increased DHE-associated (oxidant) fluorescence and increased intracellular TNF-α and IL-6, accompanied by reduced viability and progressive morphological change. Under the conditions of this study, CSE produced these effects at lower nominal puff concentrations, and in an exploratory comparison at the concentrations closest to each product’s 24 h IC_50_ its effects were larger than those of HTE. ATG5 protein levels did not differ significantly from the untreated control after exposure to either product, and the apparent increases relative to the vehicle likely reflect the anomalously low vehicle signal and the falling cell-content denominator; therefore, these data provide no evidence of an increase in ATG5 protein.

Under the experimental conditions of this study, heated tobacco aerosol extract induced smaller oxidative and inflammatory responses than cigarette smoke extract at the concentrations compared; however, the absence of chemical dose equivalence between the two products precludes any direct inference about population-level risk. The results should be regarded as preliminary observations obtained under product-specific experimental conditions, and confirmation requires chemically anchored exposure metrics, measurement of secreted mediators, flux-based assessment of autophagy and more physiologically relevant exposure models.

## Data Availability

The raw data supporting the conclusions of this article will be made available by the authors, without undue reservation.
