# Supplementary material for: Comparative oxidative and inflammatory responses to heated tobacco aerosol and cigarette smoke extracts in human lung fibroblasts
Source: Front Physiol. 2026 Oct 27;17:1902675. doi: 10.3389/fphys.2026.1902675 (PMC13747875; doi:10.3389/fphys.2026.1902675)
Supplement: Supplementary file 1 [file DataSheet1.pdf]

Supplementary Material

Comparative oxidative and inflammatory responses to heated tobacco aerosol and cigarette smoke extracts in human lung fibroblasts

Xylia A. Abelia, Ronny Lesmana, Melisa I. Barliana, Massimo Caruso, Riccardo Polosa

All statistics in this file were computed from the raw plate-reader and Odyssey data reproduced in Section S5.

Contents

S1. Preparation of extracts and working solutions (Table S1)
S2. Concentration selection and IC_{50} estimation (Tables S2–S3)
S3. Statistical analysis: approach, assumptions and results (Tables S4–S16)
S4. In-Cell Western quality control: absolute versus normalized signal (Table S17)
S5. Biological-replicate means and raw data (Tables S18–S25)

S1. Preparation of extracts and working solutions

Cigarette smoke extract (CSE) was generated from Marlboro Red cigarettes on an LM1 smoking machine (Körber, Hamburg, Germany) under the ISO 3308:2012 regimen (35 mL puff volume, 2 s puff duration, 60 s puff interval). Ten cigarettes were smoked for 8 puffs each (80 puffs in total), and the mainstream smoke was bubbled directly through 40 mL of ice-cold PBS (pH 7.4), giving a stock of 2 puffs/mL.

Heated tobacco aerosol extract (HTE) was generated from HEETS Sienna heatsticks in an IQOS 3 DUO device on an LM4E machine (Körber) under the Health Canada Intense regimen (55 mL, 2 s, 30 s) in accordance with ISO 20778:2018. Thirty heatsticks were puffed 12 times each (360 puffs in total), and the aerosol was bubbled directly through 40 mL of ice-cold PBS, giving a stock of 9 puffs/mL.

Both extracts were generated in the same laboratory (Central Laboratory, Universitas Padjadjaran) using the same PBS as capture liquid. Each extract was sterile-filtered (0.22 µm), diluted in complete culture medium to a final volume of 1 mL (Table S1), and applied to the cells within 30 min of generation. A fresh extract was prepared for each independent biological replicate.

Untreated control wells received culture medium only. The vehicle control (0 puffs/mL) consisted of 37.5% v/v PBS in culture medium (375 µL PBS + 625 µL medium) and was used as the reference for all products and concentrations. Because a single vehicle was used, its PBS content was not matched to every concentration. The highest concentrations (CSE 1.0 puffs/mL; HTE 5.0–5.5 puffs/mL) contained 50–61% v/v extract and therefore less culture medium than the vehicle. PBS is isotonic, but the dilution of medium and serum at these concentrations was not controlled for; this is acknowledged as a limitation in the main text.

The extracts were not chemically characterized (total particulate matter, nicotine, carbonyls), and pH and osmolality were not measured or standardized between products. Concentrations are therefore reported as nominal puff equivalents per mL. Because the two regimens differ in puff volume, interval and number of puffs, one CSE puff is not equivalent to one HTE puff.

Table S1. Preparation of working solutions (final volume 1 mL).

Product	Nominal concentration (puffs/mL)	Stock extract (µL)	Culture medium (µL)	Extract in medium (% v/v)
Vehicle (both products)	0	375 µL PBS	625	37.5 (PBS)
CSE	0.10	50	950	5.0
CSE	0.30	150	850	15.0
CSE	0.50	250	750	25.0
CSE	0.70	350	650	35.0
CSE	1.00	500	500	50.0
HTE	1.50	167	833	16.7
HTE	2.00	222	778	22.2
HTE	2.50	278	722	27.8
HTE	3.00	333	667	33.3
HTE	3.50	389	611	38.9
HTE	4.00	444	556	44.4
HTE	4.50	500	500	50.0
HTE	4.75	528	472	52.8
HTE	5.00	556	444	55.6
HTE	5.50	611	389	61.1

Stock concentrations: CSE 2 puffs/mL; HTE 9 puffs/mL. Stock volume = nominal concentration ÷ stock concentration × 1000 µL. HTE 1.5, 2.0, 2.5, 3.5 and 4.5 puffs/mL were used only in the cytotoxicity (dose-finding) assay; HTE 4.75 puffs/mL was used only in the mechanistic assays; HTE 3.0, 4.0, 5.0 and 5.5 puffs/mL were used in both.

S2. Concentration selection and IC_{50} estimation

Cytotoxicity (24 h, PrestoBlue) served as the dose-finding experiment. CSE was tested at 0.1–1.0 puffs/mL (five concentrations) and HTE at 1.5–5.5 puffs/mL (nine concentrations). The HTE range extends to higher nominal concentrations because HTE caused no loss of viability at 2.5 puffs/mL or below (Table S8), so higher nominal concentrations were required to reach its IC_{50}.

IC_{50} values were estimated by nonlinear least-squares regression of all biological-replicate viability values on concentration, using a normalized log-logistic model with variable slope (Top = 100%, the vehicle; Bottom = 0%). 95% confidence intervals were derived from the parameter standard errors (IC_{50} on the log scale). An unconstrained four-parameter fit was not used because, for CSE, it returned a non-physical lower asymptote.

Table S2. Dose–response parameters (24 h exposure, PrestoBlue assay).

Product	Model	IC_{50} (puffs/mL)	95% CI	Hill slope (95% CI)	R^2 (replicates / means)	Data
CSE	Normalized log-logistic, variable slope (Top = 100%, Bottom = 0%)	0.70	0.63–0.77	2.90 (1.94–3.86)	0.914 / 0.977	3 × 5 + vehicle (18 points)
HTE	Normalized log-logistic, variable slope (Top = 100%, Bottom = 0%)	4.76	4.39–5.16	3.48 (2.25–4.71)	0.809 / 0.905	3 × 9 + vehicle (30 points)

The HTE fit does not capture the slight increase in resazurin reduction at 1.5–2.0 puffs/mL (113% and 106% of vehicle; not significant, Table S8), which lowers its R^2.

The mechanistic assays used five concentrations per product, chosen to span each product's own cytotoxic range and to include each product's IC_{50} (Table S3). Expressing concentrations as fractions of the IC_{50} placed the two products on a common, product-internal scale of cytotoxic potency without requiring puff equivalence. The 24-h IC_{50} was used as a scaling anchor, not as a prediction of cell death at the shorter exposure times (90 min DHE; 4 h ICW; 10 h morphology). At the 4-h ICW time point, cell content was measured directly with CellTag 700 (Table S17): it was reduced by at most 30–34% at the highest concentrations, so the cytokine measurements were made in largely intact monolayers.

Table S3. Concentrations used in the mechanistic assays, expressed as multiples of each product's IC_{50}.

Product	Concentration (puffs/mL)	Multiple of own IC_{50}
CSE	0.1	0.14
CSE	0.3	0.43
CSE	0.5	0.71
CSE	0.7	1.00
CSE	1	1.43
HTE	3	0.63
HTE	4	0.84
HTE	4.75	1.00
HTE	5	1.05
HTE	5.5	1.16

IC_{50} values from Table S2 (CSE 0.70; HTE 4.76 puffs/mL). CSE 0.7 puffs/mL and HTE 4.75 puffs/mL are the tested concentrations closest to each product's IC_{50} and are used in the exploratory comparison in Table S16.

S3. Statistical analysis

Approach

- **Unit of analysis.** Each experiment comprised three independent biological replicates, each performed with a freshly prepared extract. Within each biological replicate, every condition was measured in technical triplicate wells. Technical replicates were averaged within each biological replicate, and all tests were performed on the three biological-replicate means (n = 3 per group). Technical replicates were never treated as independent observations.
- **Plate layout.** For the In-Cell Western (ICW) endpoints (TNF-α, IL-6, ATG5), CSE and HTE were sharing the untreated-control and vehicle wells. These endpoints were therefore analysed as a single family (vehicle plus ten treatment concentrations). DHE and PrestoBlue assays were run with their own control and vehicle wells, and were analysed per product.
- **Global test.** One-way ANOVA across the vehicle and treatment concentrations. Effect sizes are reported as η^2 and ω^2.
- **Assumptions.** Normality of residuals (Shapiro–Wilk) and homogeneity of variance (Levene's test, median-centred) were checked for every analysis family (Table S6); neither assumption was rejected for any endpoint.
- **Comparisons with control.** Each concentration was compared with the vehicle using Dunnett's two-sided many-to-one test. Adjusted p-values and 95% confidence intervals of the differences are reported. The vehicle was compared with the untreated control using Welch's t-test (Table S4). For ATG5 the untreated control was retained as the reference condition and each concentration was compared with it by the same Dunnett procedure, with the vehicle-referenced

comparisons reported alongside them (Table S13); the background-corrected 800 nm channel and the 700 nm cell-content channel were analysed in the same way (Table S17). Antimycin A was compared with the vehicle using Welch's t-test.

- **DHE.** The reagent blank (Table S22) was subtracted from every well before analysis. Blank-corrected fold-change values were normalized to the mean vehicle signal within each biological replicate, which gives the vehicle a variance of exactly zero. Inference was therefore performed on the blank-corrected fluorescence (RFU) biological-replicate means; fold changes are shown for readability.
- **Concentration trend.** Linear regression of the response on $\log_{10}$(concentration) across treatment concentrations, with Spearman's rank correlation as a distribution-free check (Table S14).
- **Comparison between products.** A parallel-line analysis (ANCOVA on $\log_{10}$ concentration with a product × concentration interaction) was used to test whether a single relative-potency factor could describe the difference between CSE and HTE (Table S15). Because parallelism was rejected for the ROS, TNF-α, IL-6 and viability curves, no single potency ratio is reported. Instead, the products were compared, in an exploratory analysis, at the tested concentrations closest to each product's own IC_{50} (CSE 0.7 vs HTE 4.75 puffs/mL; Table S16). ICW values were compared by paired t-test (both products on the same plate in each biological replicate) and DHE values by Welch's t-test (separate plates).
- **Software and reporting.** Python 3.11 with SciPy 1.17 (f_oneway, dunnett, shapiro, levene, ttest_ind, ttest_rel, linregress, spearmanr, curve_fit). Exact p-values are reported, except that Dunnett-adjusted p-values below 0.001 are given as < 0.001, because they are obtained by numerical integration and are not stable beyond that level; other p-values below 10^{-15} are shown as $< 1 \times 10^{-15}$. Significance threshold α = 0.05.

Table S4. Vehicle (37.5% v/v PBS) versus untreated control.

Endpoint	Untreated control (mean)	Vehicle (mean)	Vehicle as % of control	p (Welch t-test)	Significance
TNF-α	0.8245	0.8204	99.5	0.790	ns
IL-6	0.405	0.4288	105.9	0.134	ns
ATG5	0.555	0.2014	36.3	9.63×10^{-4}	***
ROS (DHE) (CSE)	9131	9051	99.1	0.849	ns
ROS (DHE) (HTE)	9105	9080	99.7	0.949	ns
Cell viability (CSE)	100.1	100	99.9	0.968	ns
Cell viability (HTE)	114.2	100	87.5	0.205	ns

Units: TNF-α and IL-6, 800/700 ratio; ATG5, 800/700 ratio; DHE, blank-corrected RFU; viability, % of vehicle. The vehicle did not differ from the untreated control for any endpoint except ATG5, where the vehicle signal was 36% of the untreated control (see Section S4).

Table S5. One-way ANOVA and effect sizes.

Endpoint	Family analysed	Groups	F statistic	p	η^2	ω^2
TNF-α	vehicle + 10 treatment concentrations (shared plate)	11	F(10, 22) = 505.8	$< 1 \times 10^{-15}$	0.996	0.994
IL-6	vehicle + 10 treatment concentrations (shared plate)	11	F(10, 22) = 142.0	$< 1 \times 10^{-15}$	0.985	0.977
ATG5	vehicle + 10 treatment concentrations (shared plate)	11	F(10, 22) = 3.7	0.005	0.626	0.449
ROS (DHE) (CSE)	vehicle + treatment concentrations	6	F(5, 12) = 255.6	9.40×10^{-12}	0.991	0.986
ROS (DHE) (HTE)	vehicle + treatment concentrations	6	F(5, 12) = 81.1	7.99×10^{-9}	0.971	0.957
Cell viability (CSE)	vehicle + treatment concentrations	6	F(5, 12) = 35.2	9.04×10^{-7}	0.936	0.905
Cell viability (HTE)	vehicle + treatment concentrations	10	F(9, 20) = 18.6	7.22×10^{-8}	0.893	0.841

Each group n = 3 biological replicates.

Table S6. Assumption checks on ANOVA residuals.

Endpoint	Shapiro–Wilk W	Shapiro–Wilk p	Levene W	Levene p
TNF-α	0.983	0.871	0.429	0.917
IL-6	0.957	0.209	0.328	0.964
ATG5	0.989	0.975	0.388	0.938
ROS (DHE) (CSE)	0.942	0.317	0.252	0.931
ROS (DHE) (HTE)	0.982	0.967	0.580	0.715
Cell viability (CSE)	0.960	0.601	0.816	0.561
Cell viability (HTE)	0.970	0.528	0.211	0.989

Table S7. Cell viability after 24 h CSE exposure (% of vehicle).

Concentration (puffs/mL)	Mean ± SD	Fold vs vehicle	Difference vs vehicle	95% CI of difference	Dunnett-adjusted p	Significance
0.1	98.0 ± 5.4	0.98	-2.0	-23.1 to 19.2	0.999	ns
0.3	90.1 ± 12.5	0.90	-9.9	-31.0 to 11.2	0.545	ns
0.5	68.6 ± 15.3	0.69	-31.4	-52.5 to -10.3	0.004	**
0.7	57.3 ± 5.1	0.57	-42.7	-63.9 to -21.6	< 0.001	***
1	20.4 ± 4.4	0.20	-79.6	-100.8 to -58.5	< 0.001	***

*Values are means ± SD of n = 3 independent biological replicates (each the mean of technical triplicate wells). Two-sided Dunnett test versus the vehicle control (37.5% v/v PBS). * $p < 0.05$, ** $p < 0.01$, *** $p < 0.001$; ns, not significant.*

Table S8. Cell viability after 24 h HTE exposure (% of vehicle).

Concentration (puffs/mL)	Mean ± SD	Fold vs vehicle	Difference vs vehicle	95% CI of difference	Dunnett-adjusted p	Significance
1.5	113.5 ± 15.1	1.14	13.5	-11.6 to 38.6	0.531	ns
2	106.1 ± 12.0	1.06	6.1	-19.0 to 31.2	0.983	ns
2.5	97.7 ± 8.4	0.98	-2.3	-27.4 to 22.8	1.000	ns
3	75.5 ± 14.5	0.75	-24.5	-49.6 to 0.6	0.058	ns
3.5	65.8 ± 11.6	0.66	-34.2	-59.3 to -9.1	0.005	**
4	61.6 ± 8.0	0.62	-38.4	-63.5 to -13.2	0.002	**
4.5	58.7 ± 7.3	0.59	-41.3	-66.4 to -16.2	< 0.001	***
5	49.5 ± 10.6	0.49	-50.5	-75.6 to -25.4	< 0.001	***
5.5	38.2 ± 7.5	0.38	-61.8	-86.9 to -36.7	< 0.001	***

*Values are means ± SD of n = 3 independent biological replicates (each the mean of technical triplicate wells). Two-sided Dunnett test versus the vehicle control (37.5% v/v PBS). * $p < 0.05$, ** $p < 0.01$, *** $p < 0.001$; ns, not significant.*

Table S9. Intracellular ROS (DHE) after 90 min CSE exposure (blank-corrected RFU).

Concentration (puffs/mL)	Mean ± SD	Fold vs vehicle	Difference vs vehicle	95% CI of difference	Dunnett-adjusted p	Significance
0.1	13306 ± 390	1.47	4255	1966 to 6544	< 0.001	***
0.3	18492 ± 1061	2.04	9441	7152 to 11730	< 0.001	***
0.5	24573 ± 1225	2.71	15522	13233 to 17811	< 0.001	***
0.7	28664 ± 956	3.17	19613	17324 to 21902	< 0.001	***
1	31869 ± 1317	3.52	22818	20530 to 25107	< 0.001	***
Antimycin A (positive control)	40865 ± 2260	4.51	31815	—	0.001	** (Welch t-test)

*Values are means ± SD of n = 3 independent biological replicates (each the mean of technical triplicate wells). Two-sided Dunnett test versus the vehicle control (37.5% v/v PBS). * p < 0.05, ** p < 0.01, *** p < 0.001; ns, not significant. Antimycin A (50 µM, 30 min) compared with vehicle by Welch's t-test.*

Table S10. Intracellular ROS (DHE) after 90 min HTE exposure (blank-corrected RFU).

Concentration (puffs/mL)	Mean ± SD	Fold vs vehicle	Difference vs vehicle	95% CI of difference	Dunnett-adjusted p	Significance
3	16095 ± 1044	1.77	7014	4429 to 9599	< 0.001	***
4	18140 ± 1048	2.00	9060	6475 to 11645	< 0.001	***
4.75	20992 ± 795	2.31	11912	9327 to 14497	< 0.001	***
5	23051 ± 887	2.54	13971	11386 to 16556	< 0.001	***
5.5	24805 ± 1847	2.73	15724	13139 to 18309	< 0.001	***
Antimycin A (positive control)	41521 ± 2368	4.57	32441	—	0.001	** (Welch t-test)

*Values are means ± SD of n = 3 independent biological replicates (each the mean of technical triplicate wells). Two-sided Dunnett test versus the vehicle control (37.5% v/v PBS). * p < 0.05, ** p < 0.01, *** p < 0.001; ns, not significant. Antimycin A (50 µM, 30 min) compared with vehicle by Welch's t-test.*

Table S11. Intracellular TNF-α after 4 h exposure (ICW, 800/700 ratio).

Concentration (puffs/mL)	Mean ± SD	Fold vs vehicle	Difference vs vehicle	95% CI of difference	Dunnett-adjusted p	Significance
CSE 0.1	1.065 ± 0.020	1.30	0.245	0.164 to 0.325	< 0.001	***
CSE 0.3	1.291 ± 0.020	1.57	0.471	0.390 to 0.551	< 0.001	***
CSE 0.5	1.625 ± 0.017	1.98	0.804	0.724 to 0.885	< 0.001	***
CSE 0.7	1.888 ± 0.047	2.30	1.068	0.987 to 1.148	< 0.001	***
CSE 1.0	2.154 ± 0.016	2.63	1.333	1.253 to 1.414	< 0.001	***
HTE 3.0	0.955 ± 0.026	1.16	0.135	0.055 to 0.215	< 0.001	***
HTE 4.0	1.207 ± 0.027	1.47	0.387	0.306 to 0.467	< 0.001	***
HTE 4.75	1.454 ± 0.018	1.77	0.633	0.553 to 0.714	< 0.001	***
HTE 5.0	1.690 ± 0.062	2.06	0.869	0.789 to 0.950	< 0.001	***
HTE 5.5	1.924 ± 0.052	2.34	1.103	1.023 to 1.184	< 0.001	***

*Values are means ± SD of n = 3 independent biological replicates (each the mean of technical triplicate wells). Two-sided Dunnett test versus the vehicle control (37.5% v/v PBS). * p < 0.05, ** p < 0.01, *** p < 0.001; ns, not significant. CSE and HTE concentrations were analysed as one family because they shared control and vehicle wells.*

Table S12. Intracellular IL-6 after 4 h exposure (ICW, 800/700 ratio).

Concentration (puffs/mL)	Mean ± SD	Fold vs vehicle	Difference vs vehicle	95% CI of difference	Dunnett-adjusted p	Significance
CSE 0.1	0.563 ± 0.026	1.32	0.135	0.012 to 0.257	0.026	*
CSE 0.3	0.832 ± 0.069	1.94	0.403	0.281 to 0.526	< 0.001	***
CSE 0.5	1.036 ± 0.034	2.42	0.607	0.485 to 0.730	< 0.001	***
CSE 0.7	1.312 ± 0.063	3.06	0.883	0.761 to 1.006	< 0.001	***
CSE 1.0	1.393 ± 0.084	3.25	0.964	0.842 to 1.086	< 0.001	***
HTE 3.0	0.447 ± 0.017	1.04	0.018	-0.104 to 0.140	1.000	ns
HTE 4.0	0.587 ± 0.032	1.37	0.158	0.036 to 0.280	0.007	**
HTE 4.75	0.830 ± 0.043	1.94	0.401	0.279 to 0.524	< 0.001	***
HTE 5.0	0.989 ± 0.034	2.31	0.560	0.438 to 0.682	< 0.001	***
HTE 5.5	1.264 ± 0.079	2.95	0.836	0.713 to 0.958	< 0.001	***

*Values are means ± SD of n = 3 independent biological replicates (each the mean of technical triplicate wells). Two-sided Dunnett test versus the vehicle control (37.5% v/v PBS). * p < 0.05, ** p < 0.01, *** p < 0.001; ns, not significant. CSE and HTE concentrations were analysed as one family because they shared control and vehicle wells.*

Table S13. ATG5 after 4 h exposure (ICW, 800/700 ratio), compared with the vehicle and with the untreated control.

Concentration (puffs/mL)	Mean ± SD	Fold vs vehicle	Difference vs vehicle	95% CI of difference	Dunnett-adjusted p	Significance	Dunnett p vs untreated control	Significance vs untreated
CSE 0.1	0.381 ± 0.064	2.00	0.180	-0.130 to 0.489	0.466	ns	0.502	ns
CSE 0.3	0.401 ± 0.083	2.06	0.200	-0.110 to 0.509	0.353	ns	0.633	ns
CSE 0.5	0.529 ± 0.185	2.85	0.327	0.018 to 0.637	0.035	*	1.000	ns
CSE 0.7	0.642 ± 0.166	3.24	0.440	0.131 to 0.750	0.003	**	0.970	ns
CSE 1.0	0.755 ± 0.209	3.88	0.554	0.244 to 0.863	< 0.001	***	0.352	ns
HTE 3.0	0.463 ± 0.096	2.44	0.262	-0.047 to 0.572	0.125	ns	0.959	ns
HTE 4.0	0.492 ± 0.117	2.52	0.290	-0.019 to 0.600	0.074	ns	0.996	ns
HTE 4.75	0.511 ± 0.118	2.74	0.310	0.000 to 0.619	0.050	ns	1.000	ns
HTE 5.0	0.516 ± 0.107	2.73	0.314	0.005 to 0.624	0.046	*	1.000	ns
HTE 5.5	0.542 ± 0.116	2.85	0.340	0.031 to 0.650	0.026	*	1.000	ns

Compared with the vehicle, several concentrations appear increased. However, the vehicle itself was 64% below the untreated control (Table S4), and no concentration of either product differed from the untreated control. See Section S4. Fold vs vehicle values are means of the per-replicate ratios (treatment ÷ vehicle within each biological replicate) and therefore differ from the ratio of the group means when the vehicle signal varies between replicates. Adjusted p-values are shown to three decimal places; HTE 4.75 puffs/mL is marked ns because its unrounded value did not fall below α = 0.05.

Table S14. Concentration–response trend across treatment concentrations ($\log_{10}$ scale).

Endpoint	Product	Slope per $\log_{10}$ unit	p (slope)	R^2	Spearman ρ	p (Spearman)
Cell viability	CSE	-70	5.44×10^{-5}	0.73	-0.93	6.49×10^{-7}
Cell viability	HTE	-136	5.25×10^{-12}	0.86	-0.93	3.13×10^{-12}
ROS (DHE fold)	CSE	2.09	5.28×10^{-10}	0.95	0.98	8.65×10^{-11}
ROS (DHE fold)	HTE	3.62	7.57×10^{-8}	0.90	0.98	8.65×10^{-11}
TNF-α	CSE	1.08	3.18×10^{-8}	0.91	0.98	8.65×10^{-11}

Endpoint	Product	Slope per $\log_{10}$ unit	p (slope)	R^2	Spearman ρ	p (Spearman)
TNF-α	HTE	3.54	4.43×10^{-8}	0.91	0.98	8.65×10^{-11}
IL-6	CSE	0.858	7.73×10^{-9}	0.93	0.96	1.43×10^{-8}
IL-6	HTE	2.91	1.09×10^{-6}	0.85	0.98	8.65×10^{-11}
ATG5 (% control)	CSE	66.2	0.006	0.46	0.76	9.19×10^{-4}
ATG5 (% control)	HTE	49.7	0.330	0.07	0.25	0.367

ATG5 trends were computed on values expressed as a percentage of the untreated control, the same normalization as Figure 9 and Table S18. Trend analyses use the three biological-replicate means at each treatment concentration (CSE, 15 points; HTE, 15 points; HTE viability, 27 points). DHE trends use fold-change values.

Table S15. Test of parallelism between CSE and HTE concentration–response lines ($\log_{10}$ scale).

Endpoint	F (product × log concentration)	p	Parallel lines?
ROS (DHE fold)	12.51	0.002	No
TNF-α	50.92	1.41×10^{-7}	No
IL-6	44.24	4.68×10^{-7}	No
Cell viability	15.96	2.86×10^{-4}	No
ATG5	0.06	0.804	Yes

F-test with 1 and 26 (viability: 38) degrees of freedom. When lines are not parallel, the ratio of concentrations producing equal responses depends on the response level chosen, so a single relative-potency factor is not defensible. The products are therefore compared, in an exploratory analysis, at the tested concentrations closest to each product's own IC_{50} (Table S16) rather than by a fold-difference in potency.

Table S16. Exploratory comparison of the two products at the tested concentrations closest to each product's own 24 h IC_{50} (CSE 0.7 vs HTE 4.75 puffs/mL). Values are fold change versus vehicle.

Endpoint	Signal	CSE (mean ± SD)	HTE (mean ± SD)	Test	p	Significance
TNF-α	ratio (800/700)	2.30 ± 0.01	1.77 ± 0.06	paired t (same plate per biological replicate)	0.005	**
TNF-α	absolute 800 nm	1.80 ± 0.06	1.55 ± 0.09	paired t (same plate per biological replicate)	0.087	ns
IL-6	ratio (800/700)	3.06 ± 0.17	1.94 ± 0.12	paired t (same plate per biological replicate)	0.015	*
IL-6	absolute 800 nm	2.36 ± 0.09	1.70 ± 0.08	paired t (same plate per biological replicate)	0.023	*
ATG5	ratio (800/700)	3.24 ± 0.76	2.74 ± 1.31	paired t (same plate per biological replicate)	0.620	ns
ATG5	absolute 800 nm	2.30 ± 0.42	2.37 ± 1.15	paired t (same plate per biological replicate)	0.927	ns
ROS (DHE)	fold vs own vehicle	3.17 ± 0.04	2.31 ± 0.08	Welch t (separate plates)	3.56×10^{-4}	***

n = 3 biological replicates. At these concentrations the HTE wells contained a larger extract fraction, and therefore less culture medium, than the CSE wells (52.8% vs 35% v/v), and the single 37.5% PBS vehicle matched neither; this unmatched medium dilution is a limitation of the comparison. Equal cytotoxicity at the 90 min DHE and 4 h ICW time points was not tested. Fold changes are means of the per-replicate ratios; for the limitations of the ATG5 vehicle reference, see Section S4.

S4. In-Cell Western quality control: absolute versus normalized signal

ICW signals were normalized to the CellTag 700 channel, which reports total cell content per well. Because exposure reduces cell number, a normalized ratio can rise partly because its denominator falls. Table S17 therefore reports, for every concentration, the background-corrected 800 nm signal (target protein), the 700 nm signal (cell content) and the 800/700 ratio, each as fold change versus the vehicle.

- **TNF-α and IL-6** increased in the absolute 800 nm channel (up to 1.8-fold and 2.5-fold for CSE; 1.9-fold and 2.3-fold for HTE), so these responses are not normalization artefacts. Cell content fell to 0.70–0.81 of the vehicle at the highest concentrations, so at those concentrations the ratio exceeds the absolute fold change by about 20–45%. Both readouts are reported in the main text.
- **ATG5** did not exceed the untreated-control level in the absolute channel at any concentration, and its apparent rise in the ratio tracks the fall in cell content. In addition, the vehicle wells of the ATG5 plate carried an absolute signal 2.9-fold lower than the untreated-control wells at equal cell content (Table S17), whereas the vehicle and untreated control did not differ for TNF-α or IL-6. No concentration of either product differed from the untreated control (Table S13). These data therefore provide no evidence of an increase in ATG5 protein, and the ATG5 results are not interpreted in terms of autophagy.

Table S17. Absolute target signal, cell content and normalized ratio for the ICW endpoints (fold change versus vehicle).

Endpoint	Group	Absolute 800 nm (fold)	Significance	Cell content, 700 nm (fold)	Significance	800/700 ratio (fold)
TNF-α	CSE 0.1	1.29	***	1.00	ns	1.30
TNF-α	CSE 0.3	1.37	***	0.87	**	1.57
TNF-α	CSE 0.5	1.66	***	0.84	***	1.98
TNF-α	CSE 0.7	1.80	***	0.78	***	2.30
TNF-α	CSE 1.0	1.83	***	0.70	***	2.63
TNF-α	HTE 3.0	1.15	ns	0.99	ns	1.16
TNF-α	HTE 4.0	1.36	***	0.93	ns	1.47
TNF-α	HTE 4.75	1.55	***	0.87	**	1.77
TNF-α	HTE 5.0	1.75	***	0.85	***	2.06
TNF-α	HTE 5.5	1.91	***	0.81	***	2.34
TNF-α	Untreated control	0.99		0.98		1.01
IL-6	CSE 0.1	1.22	ns	0.93	ns	1.32
IL-6	CSE 0.3	1.75	***	0.90	**	1.94
IL-6	CSE 0.5	1.99	***	0.82	***	2.42
IL-6	CSE 0.7	2.36	***	0.77	***	3.06
IL-6	CSE 1.0	2.45	***	0.75	***	3.25
IL-6	HTE 3.0	1.02	ns	0.98	ns	1.04
IL-6	HTE 4.0	1.26	ns	0.92	*	1.37
IL-6	HTE 4.75	1.70	***	0.88	**	1.94
IL-6	HTE 5.0	1.96	***	0.85	***	2.31
IL-6	HTE 5.5	2.27	***	0.77	***	2.95
IL-6	Untreated control	0.91		0.97		0.95
ATG5	CSE 0.1	2.04	ns	1.01	ns	2.00
ATG5	CSE 0.3	1.90	ns	0.92	ns	2.06
ATG5	CSE 0.5	2.37	ns	0.84	***	2.85
ATG5	CSE 0.7	2.30	*	0.72	***	3.24
ATG5	CSE 1.0	2.56	*	0.66	***	3.88

Endpoint	Group	Absolute 800 nm (fold)	Significance	Cell content, 700 nm (fold)	Significance	800/700 ratio (fold)
ATG5	HTE 3.0	2.46	*	0.99	ns	2.44
ATG5	HTE 4.0	2.32	*	0.91	*	2.52
ATG5	HTE 4.75	2.37	*	0.87	***	2.74
ATG5	HTE 5.0	2.07	ns	0.76	***	2.73
ATG5	HTE 5.5	1.89	ns	0.67	***	2.85
ATG5	Untreated control	2.92		1.02		2.86

Dunnett test versus vehicle on the corresponding channel; n = 3 biological replicates. The untreated-control row is shown for reference and was not included in the Dunnett family. Fold changes are means of the per-replicate ratios (value ÷ vehicle within each biological replicate).

S5. Biological-replicate means and raw data

Tables S18–S25 contain all data underlying the figures and the statistics above, including well-level values. They supersede the supplementary tables submitted with the original version of the manuscript, including their statistical output.

Table S18. Biological-replicate means for every endpoint and group. DHE values are blank-corrected. ATG5 values are expressed relative to the mean untreated-control ratio across the three biological replicates; Figure 9 is plotted from these values.

Endpoint	Group	R1	R2	R3	Mean	SD	SEM
Cell viability (%)	Untreated control (CSE plate)	101.3	103.0	96.1	100.1	3.6	2.1
Cell viability (%)	Vehicle, 0 puff/mL (CSE plate)	101.0	102.9	96.1	100.0	3.5	2.0
Cell viability (%)	CSE 0.1	91.8	100.6	101.7	98.0	5.4	3.1
Cell viability (%)	CSE 0.3	75.9	95.0	99.4	90.1	12.5	7.2
Cell viability (%)	CSE 0.5	84.4	53.7	67.7	68.6	15.3	8.9
Cell viability (%)	CSE 0.7	61.5	51.6	58.7	57.3	5.1	3.0
Cell viability (%)	CSE 1	25.4	17.9	17.8	20.4	4.4	2.5
Cell viability (%)	Untreated control (HTE plate)	111.6	102.1	128.9	114.2	13.6	7.8
Cell viability (%)	Vehicle, 0 puff/mL (HTE plate)	99.1	104.7	96.2	100.0	4.3	2.5
Cell viability (%)	HTE 1.5	130.9	103.2	106.4	113.5	15.1	8.7
Cell viability (%)	HTE 2	117.4	107.3	93.5	106.1	12.0	6.9
Cell viability (%)	HTE 2.5	98.4	89.0	105.7	97.7	8.4	4.8
Cell viability (%)	HTE 3	88.9	77.4	60.1	75.5	14.5	8.4
Cell viability (%)	HTE 3.5	52.5	73.2	71.8	65.8	11.6	6.7
Cell viability (%)	HTE 4	57.4	56.6	70.9	61.6	8.0	4.6
Cell viability (%)	HTE 4.5	53.8	67.1	55.2	58.7	7.3	4.2
Cell viability (%)	HTE 5	54.9	56.2	37.2	49.5	10.6	6.1
Cell viability (%)	HTE 5.5	34.5	46.8	33.3	38.2	7.5	4.3
ROS (DHE, RFU)	Untreated control (CSE plate)	9352	8515	9526	9131	540	312
ROS (DHE, RFU)	Vehicle, 0 puff/mL (CSE plate)	9140	8595	9417	9051	418	242
ROS (DHE, RFU)	CSE 0.1	13390	12881	13647	13306	390	225
ROS (DHE, RFU)	CSE 0.3	18045	17727	19703	18492	1061	613

Endpoint	Group	R1	R2	R3	Mean	SD	SEM
ROS (DHE, RFU)	CSE 0.5	25335	23159	25224	24573	1225	707
ROS (DHE, RFU)	CSE 0.7	28987	27588	29417	28664	956	552
ROS (DHE, RFU)	CSE 1	32811	30364	32432	31869	1317	761
ROS (DHE, RFU)	Antimycin A (CSE plate)	40693	38696	43206	40865	2260	1305
ROS (DHE, RFU)	Untreated control (HTE plate)	8899	9680	8736	9105	504	291
ROS (DHE, RFU)	Vehicle, 0 puff/mL (HTE plate)	9005	9481	8755	9080	369	213
ROS (DHE, RFU)	HTE 3	15949	17204	15131	16095	1044	603
ROS (DHE, RFU)	HTE 4	17853	19302	17266	18140	1048	605
ROS (DHE, RFU)	HTE 4.75	20203	21794	20979	20992	795	459
ROS (DHE, RFU)	HTE 5	23420	23695	22039	23051	887	512
ROS (DHE, RFU)	HTE 5.5	25180	26435	22799	24805	1847	1066
ROS (DHE, RFU)	Antimycin A (HTE plate)	41808	43733	39023	41521	2368	1367
TNF-α (800/700)	Untreated control	0.8258	0.8424	0.8054	0.8245	0.0185	0.0107
TNF-α (800/700)	Vehicle (0 puff/mL)	0.8189	0.8041	0.8382	0.8204	0.0171	0.0099
TNF-α (800/700)	CSE 0.1	1.0740	1.0789	1.0419	1.0649	0.0201	0.0116
TNF-α (800/700)	CSE 0.3	1.3145	1.2790	1.2797	1.2910	0.0203	0.0117
TNF-α (800/700)	CSE 0.5	1.6076	1.6424	1.6238	1.6246	0.0174	0.0101
TNF-α (800/700)	CSE 0.7	1.8799	1.8455	1.9386	1.8880	0.0471	0.0272
TNF-α (800/700)	CSE 1.0	2.1353	2.1662	2.1599	2.1538	0.0163	0.0094
TNF-α (800/700)	HTE 3.0	0.9257	0.9654	0.9751	0.9554	0.0262	0.0151
TNF-α (800/700)	HTE 4.0	1.2369	1.1841	1.2001	1.2070	0.0271	0.0156
TNF-α (800/700)	HTE 4.75	1.4579	1.4691	1.4347	1.4539	0.0176	0.0101
TNF-α (800/700)	HTE 5.0	1.6541	1.7615	1.6532	1.6896	0.0622	0.0359
TNF-α (800/700)	HTE 5.5	1.8668	1.9346	1.9692	1.9235	0.0521	0.0301
IL-6 (800/700)	Untreated control	0.4205	0.3986	0.3959	0.4050	0.0135	0.0078
IL-6 (800/700)	Vehicle (0 puff/mL)	0.4308	0.4110	0.4447	0.4288	0.0169	0.0098
IL-6 (800/700)	CSE 0.1	0.5728	0.5833	0.5344	0.5635	0.0257	0.0149
IL-6 (800/700)	CSE 0.3	0.7600	0.8387	0.8974	0.8320	0.0690	0.0398
IL-6 (800/700)	CSE 0.5	1.0302	1.0725	1.0053	1.0360	0.0340	0.0196
IL-6 (800/700)	CSE 0.7	1.3842	1.2709	1.2815	1.3122	0.0626	0.0361
IL-6 (800/700)	CSE 1.0	1.4891	1.3401	1.3493	1.3928	0.0835	0.0482
IL-6 (800/700)	HTE 3.0	0.4650	0.4446	0.4308	0.4468	0.0172	0.0100
IL-6 (800/700)	HTE 4.0	0.5995	0.6114	0.5501	0.5870	0.0325	0.0188
IL-6 (800/700)	HTE 4.75	0.7816	0.8476	0.8611	0.8301	0.0425	0.0245
IL-6 (800/700)	HTE 5.0	0.9619	0.9775	1.0274	0.9890	0.0342	0.0198
IL-6 (800/700)	HTE 5.5	1.3551	1.2173	1.2209	1.2644	0.0785	0.0454
ATG5 (% of untreated control)	Untreated control	96.2	93.5	110.3	100.0	9.0	5.2
ATG5 (% of untreated control)	Vehicle (0 puff/mL)	45.5	27.6	35.8	36.3	8.9	5.2
ATG5 (% of untreated control)	CSE 0.1	56.4	70.1	79.5	68.7	11.6	6.7
ATG5 (% of untreated control)	CSE 0.3	66.5	61.1	89.3	72.3	15.0	8.6

Endpoint	Group	R1	R2	R3	Mean	SD	SEM
ATG5 (% of untreated control)	CSE 0.5	88.0	131.7	66.1	95.3	33.4	19.3
ATG5 (% of untreated control)	CSE 0.7	116.4	85.4	145.0	115.6	29.8	17.2
ATG5 (% of untreated control)	CSE 1.0	165.2	149.5	93.4	136.0	37.7	21.8
ATG5 (% of untreated control)	HTE 3.0	85.5	99.6	65.4	83.5	17.2	9.9
ATG5 (% of untreated control)	HTE 4.0	106.4	94.0	65.3	88.6	21.1	12.2
ATG5 (% of untreated control)	HTE 4.75	77.6	116.6	82.0	92.1	21.3	12.3
ATG5 (% of untreated control)	HTE 5.0	89.7	113.6	75.5	92.9	19.3	11.1
ATG5 (% of untreated control)	HTE 5.5	100.9	116.7	75.3	97.6	20.9	12.1

Table S19. Cell viability, well-level raw data (PrestoBlue, 24 h).

Product	Condition	Concentration	Bio Rep	Technical Rep	Raw A570	Raw A600	A570−A600	Blank-corrected absorbance	Cell viability (%)
CSE	C	N/A	R1	T1	0.9973	0.1948	0.8025	0.7545	101.69
CSE	C	N/A	R1	T2	0.9832	0.1971	0.7861	0.7381	99.48
CSE	C	N/A	R1	T3	1.0124	0.2024	0.8100	0.7620	102.69
CSE	C	N/A	R2	T1	0.9777	0.1543	0.8233	0.7753	104.49
CSE	C	N/A	R2	T2	0.9697	0.1528	0.8169	0.7689	103.62
CSE	C	N/A	R2	T3	0.9454	0.1497	0.7956	0.7476	100.76
CSE	C	N/A	R3	T1	0.8981	0.1324	0.7656	0.7176	96.72
CSE	C	N/A	R3	T2	0.9058	0.1391	0.7667	0.7187	96.86
CSE	C	N/A	R3	T3	0.8917	0.1404	0.7513	0.7033	94.79
CSE	0 puff	0	R1	T1	0.9385	0.1450	0.7935	0.7455	100.47
CSE	0 puff	0	R1	T2	0.9489	0.1416	0.8073	0.7593	102.34
CSE	0 puff	0	R1	T3	0.9351	0.1437	0.7913	0.7433	100.18
CSE	0 puff	0	R2	T1	0.9661	0.1590	0.8071	0.7591	102.31
CSE	0 puff	0	R2	T2	0.9745	0.1508	0.8237	0.7757	104.54
CSE	0 puff	0	R2	T3	0.9624	0.1596	0.8028	0.7548	101.72
CSE	0 puff	0	R3	T1	0.9348	0.1802	0.7546	0.7066	95.23
CSE	0 puff	0	R3	T2	0.9392	0.1873	0.7519	0.7039	94.86
CSE	0 puff	0	R3	T3	0.9674	0.1896	0.7778	0.7298	98.35
CSE	Treatment	0.1	R1	T1	0.9190	0.1883	0.7307	0.6827	92.01
CSE	Treatment	0.1	R1	T2	0.9113	0.1896	0.7218	0.6738	90.81
CSE	Treatment	0.1	R1	T3	0.9178	0.1825	0.7353	0.6873	92.62
CSE	Treatment	0.1	R2	T1	0.9857	0.1819	0.8037	0.7557	101.85
CSE	Treatment	0.1	R2	T2	0.9774	0.1892	0.7881	0.7401	99.75
CSE	Treatment	0.1	R2	T3	0.9812	0.1890	0.7922	0.7442	100.30
CSE	Treatment	0.1	R3	T1	0.9654	0.1571	0.8083	0.7603	102.47
CSE	Treatment	0.1	R3	T2	0.9539	0.1490	0.8049	0.7569	102.01
CSE	Treatment	0.1	R3	T3	0.9497	0.1551	0.7946	0.7466	100.62
CSE	Treatment	0.3	R1	T1	0.7770	0.1547	0.6223	0.5743	77.40
CSE	Treatment	0.3	R1	T2	0.7585	0.1589	0.5996	0.5516	74.34

Product	Condition	Concentration	Bio Rep	Technical Rep	Raw A570	Raw A600	A570−A600	Blank-corrected absorbance	Cell viability (%)
CSE	Treatment	0.3	R1	T3	0.7641	0.1531	0.6110	0.5630	75.88
CSE	Treatment	0.3	R2	T1	0.8867	0.1385	0.7483	0.7003	94.37
CSE	Treatment	0.3	R2	T2	0.9092	0.1416	0.7676	0.7196	96.98
CSE	Treatment	0.3	R2	T3	0.8885	0.1451	0.7434	0.6954	93.72
CSE	Treatment	0.3	R3	T1	0.9148	0.1377	0.7770	0.7290	98.25
CSE	Treatment	0.3	R3	T2	0.9267	0.1448	0.7819	0.7339	98.91
CSE	Treatment	0.3	R3	T3	0.9366	0.1390	0.7976	0.7496	101.03
CSE	Treatment	0.5	R1	T1	0.8619	0.1953	0.6666	0.6186	83.37
CSE	Treatment	0.5	R1	T2	0.8508	0.1849	0.6658	0.6178	83.26
CSE	Treatment	0.5	R1	T3	0.8793	0.1895	0.6898	0.6418	86.49
CSE	Treatment	0.5	R2	T1	0.5882	0.1457	0.4424	0.3944	53.16
CSE	Treatment	0.5	R2	T2	0.5917	0.1467	0.4450	0.3970	53.51
CSE	Treatment	0.5	R2	T3	0.6036	0.1507	0.4529	0.4049	54.56
CSE	Treatment	0.5	R3	T1	0.7445	0.1900	0.5545	0.5065	68.26
CSE	Treatment	0.5	R3	T2	0.7502	0.1937	0.5565	0.5085	68.53
CSE	Treatment	0.5	R3	T3	0.7274	0.1877	0.5398	0.4918	66.28
CSE	Treatment	0.7	R1	T1	0.6663	0.1566	0.5097	0.4617	62.22
CSE	Treatment	0.7	R1	T2	0.6526	0.1513	0.5013	0.4533	61.09
CSE	Treatment	0.7	R1	T3	0.6501	0.1478	0.5023	0.4543	61.22
CSE	Treatment	0.7	R2	T1	0.6351	0.2030	0.4320	0.3840	51.76
CSE	Treatment	0.7	R2	T2	0.6291	0.1960	0.4330	0.3850	51.89
CSE	Treatment	0.7	R2	T3	0.6350	0.2080	0.4270	0.3790	51.08
CSE	Treatment	0.7	R3	T1	0.6139	0.1403	0.4736	0.4256	57.36
CSE	Treatment	0.7	R3	T2	0.6217	0.1387	0.4830	0.4350	58.63
CSE	Treatment	0.7	R3	T3	0.6356	0.1415	0.4941	0.4461	60.13
CSE	Treatment	1	R1	T1	0.4375	0.1966	0.2409	0.1929	26.00
CSE	Treatment	1	R1	T2	0.4399	0.2040	0.2359	0.1879	25.33
CSE	Treatment	1	R1	T3	0.4316	0.1991	0.2325	0.1845	24.86
CSE	Treatment	1	R2	T1	0.3424	0.1588	0.1836	0.1356	18.27
CSE	Treatment	1	R2	T2	0.3303	0.1506	0.1798	0.1318	17.76
CSE	Treatment	1	R2	T3	0.3326	0.1526	0.1799	0.1319	17.78
CSE	Treatment	1	R3	T1	0.3735	0.1912	0.1822	0.1342	18.09
CSE	Treatment	1	R3	T2	0.3624	0.1859	0.1765	0.1285	17.31
CSE	Treatment	1	R3	T3	0.3738	0.1930	0.1808	0.1328	17.89
HTE	C	N/A	R1	T1	1.0952	0.1919	0.9033	0.8523	111.41
HTE	C	N/A	R1	T2	1.0963	0.2007	0.8956	0.8446	110.40
HTE	C	N/A	R1	T3	1.1145	0.1989	0.9156	0.8646	113.02
HTE	C	N/A	R2	T1	1.0418	0.1945	0.8473	0.7963	104.09
HTE	C	N/A	R2	T2	1.0163	0.1982	0.8181	0.7671	100.27
HTE	C	N/A	R2	T3	1.0254	0.1936	0.8317	0.7807	102.06
HTE	C	N/A	R3	T1	1.2057	0.1663	1.0394	0.9884	129.20
HTE	C	N/A	R3	T2	1.2087	0.1624	1.0463	0.9953	130.10

Product	Condition	Concentration	Bio Rep	Technical Rep	Raw A570	Raw A600	A570−A600	Blank-corrected absorbance	Cell viability (%)
HTE	C	N/A	R3	T3	1.1855	0.1587	1.0267	0.9757	127.55
HTE	0 puff	0	R1	T1	0.9378	0.1395	0.7984	0.7474	97.69
HTE	0 puff	0	R1	T2	0.9489	0.1363	0.8126	0.7616	99.56
HTE	0 puff	0	R1	T3	0.9553	0.1387	0.8166	0.7656	100.07
HTE	0 puff	0	R2	T1	1.0388	0.1978	0.8410	0.7900	103.26
HTE	0 puff	0	R2	T2	1.0662	0.2039	0.8623	0.8113	106.05
HTE	0 puff	0	R2	T3	1.0494	0.1966	0.8528	0.8018	104.80
HTE	0 puff	0	R3	T1	0.9561	0.1669	0.7892	0.7382	96.50
HTE	0 puff	0	R3	T2	0.9419	0.1676	0.7743	0.7233	94.55
HTE	0 puff	0	R3	T3	0.9727	0.1758	0.7970	0.7460	97.51
HTE	Treatment	1.5	R1	T1	1.2278	0.1722	1.0556	1.0046	131.32
HTE	Treatment	1.5	R1	T2	1.2100	0.1669	1.0431	0.9921	129.68
HTE	Treatment	1.5	R1	T3	1.2242	0.1664	1.0578	1.0068	131.60
HTE	Treatment	1.5	R2	T1	0.9825	0.1546	0.8280	0.7770	101.56
HTE	Treatment	1.5	R2	T2	1.0005	0.1582	0.8423	0.7913	103.44
HTE	Treatment	1.5	R2	T3	1.0090	0.1579	0.8511	0.8001	104.58
HTE	Treatment	1.5	R3	T1	1.0648	0.2085	0.8563	0.8053	105.27
HTE	Treatment	1.5	R3	T2	1.0671	0.2024	0.8648	0.8138	106.37
HTE	Treatment	1.5	R3	T3	1.0722	0.1984	0.8737	0.8227	107.55
HTE	Treatment	2	R1	T1	1.1098	0.1762	0.9336	0.8826	115.37
HTE	Treatment	2	R1	T2	1.1405	0.1783	0.9622	0.9112	119.11
HTE	Treatment	2	R1	T3	1.1202	0.1683	0.9519	0.9009	117.76
HTE	Treatment	2	R2	T1	0.9939	0.1377	0.8562	0.8052	105.26
HTE	Treatment	2	R2	T2	1.0204	0.1417	0.8787	0.8277	108.20
HTE	Treatment	2	R2	T3	1.0198	0.1383	0.8815	0.8305	108.57
HTE	Treatment	2	R3	T1	0.9274	0.1663	0.7610	0.7100	92.82
HTE	Treatment	2	R3	T2	0.9365	0.1603	0.7763	0.7253	94.81
HTE	Treatment	2	R3	T3	0.9225	0.1605	0.7620	0.7110	92.94
HTE	Treatment	2.5	R1	T1	0.9750	0.1720	0.8030	0.7520	98.30
HTE	Treatment	2.5	R1	T2	0.9517	0.1638	0.7879	0.7369	96.32
HTE	Treatment	2.5	R1	T3	0.9869	0.1662	0.8206	0.7696	100.61
HTE	Treatment	2.5	R2	T1	0.8881	0.1481	0.7400	0.6890	90.07
HTE	Treatment	2.5	R2	T2	0.8811	0.1569	0.7241	0.6731	87.99
HTE	Treatment	2.5	R2	T3	0.8873	0.1548	0.7324	0.6814	89.07
HTE	Treatment	2.5	R3	T1	1.0275	0.1637	0.8638	0.8128	106.25
HTE	Treatment	2.5	R3	T2	1.0051	0.1605	0.8447	0.7937	103.75
HTE	Treatment	2.5	R3	T3	1.0377	0.1666	0.8711	0.8201	107.21
HTE	Treatment	3	R1	T1	0.9369	0.1887	0.7483	0.6973	91.15
HTE	Treatment	3	R1	T2	0.9129	0.1877	0.7252	0.6742	88.14
HTE	Treatment	3	R1	T3	0.9017	0.1825	0.7192	0.6682	87.35
HTE	Treatment	3	R2	T1	0.7726	0.1428	0.6298	0.5788	75.66
HTE	Treatment	3	R2	T2	0.7927	0.1474	0.6452	0.5942	77.68

Product	Condition	Concentration	Bio Rep	Technical Rep	Raw A570	Raw A600	A570−A600	Blank-corrected absorbance	Cell viability (%)
HTE	Treatment	3	R2	T3	0.7952	0.1406	0.6546	0.6036	78.91
HTE	Treatment	3	R3	T1	0.6673	0.1657	0.5016	0.4506	58.90
HTE	Treatment	3	R3	T2	0.6862	0.1710	0.5153	0.4643	60.69
HTE	Treatment	3	R3	T3	0.6855	0.1690	0.5165	0.4655	60.85
HTE	Treatment	3.5	R1	T1	0.5915	0.1427	0.4488	0.3978	52.00
HTE	Treatment	3.5	R1	T2	0.5904	0.1391	0.4514	0.4004	52.33
HTE	Treatment	3.5	R1	T3	0.5989	0.1416	0.4573	0.4063	53.11
HTE	Treatment	3.5	R2	T1	0.8066	0.1999	0.6067	0.5557	72.64
HTE	Treatment	3.5	R2	T2	0.8193	0.2020	0.6173	0.5663	74.02
HTE	Treatment	3.5	R2	T3	0.8130	0.2048	0.6082	0.5572	72.84
HTE	Treatment	3.5	R3	T1	0.7970	0.1943	0.6027	0.5517	72.12
HTE	Treatment	3.5	R3	T2	0.8082	0.2048	0.6035	0.5525	72.22
HTE	Treatment	3.5	R3	T3	0.7975	0.2036	0.5938	0.5428	70.96
HTE	Treatment	4	R1	T1	0.6995	0.2029	0.4966	0.4456	58.25
HTE	Treatment	4	R1	T2	0.6845	0.1969	0.4876	0.4366	57.07
HTE	Treatment	4	R1	T3	0.6877	0.2018	0.4859	0.4349	56.85
HTE	Treatment	4	R2	T1	0.6287	0.1509	0.4778	0.4268	55.79
HTE	Treatment	4	R2	T2	0.6448	0.1590	0.4859	0.4349	56.85
HTE	Treatment	4	R2	T3	0.6488	0.1599	0.4890	0.4380	57.25
HTE	Treatment	4	R3	T1	0.7512	0.1694	0.5818	0.5308	69.39
HTE	Treatment	4	R3	T2	0.7718	0.1712	0.6006	0.5496	71.84
HTE	Treatment	4	R3	T3	0.7615	0.1637	0.5978	0.5468	71.47
HTE	Treatment	4.5	R1	T1	0.6273	0.1716	0.4558	0.4048	52.91
HTE	Treatment	4.5	R1	T2	0.6313	0.1692	0.4622	0.4112	53.75
HTE	Treatment	4.5	R1	T3	0.6380	0.1678	0.4702	0.4192	54.80
HTE	Treatment	4.5	R2	T1	0.7059	0.1501	0.5558	0.5048	65.99
HTE	Treatment	4.5	R2	T2	0.7108	0.1511	0.5597	0.5087	66.50
HTE	Treatment	4.5	R2	T3	0.7239	0.1475	0.5764	0.5254	68.67
HTE	Treatment	4.5	R3	T1	0.6306	0.1609	0.4696	0.4186	54.72
HTE	Treatment	4.5	R3	T2	0.6310	0.1573	0.4737	0.4227	55.25
HTE	Treatment	4.5	R3	T3	0.6371	0.1597	0.4774	0.4264	55.74
HTE	Treatment	5	R1	T1	0.6113	0.1383	0.4731	0.4221	55.17
HTE	Treatment	5	R1	T2	0.6085	0.1339	0.4746	0.4236	55.37
HTE	Treatment	5	R1	T3	0.6009	0.1346	0.4663	0.4153	54.28
HTE	Treatment	5	R2	T1	0.6263	0.1512	0.4751	0.4241	55.44
HTE	Treatment	5	R2	T2	0.6386	0.1566	0.4820	0.4310	56.34
HTE	Treatment	5	R2	T3	0.6466	0.1603	0.4863	0.4353	56.90
HTE	Treatment	5	R3	T1	0.5088	0.1720	0.3368	0.2858	37.36
HTE	Treatment	5	R3	T2	0.5066	0.1678	0.3388	0.2878	37.62
HTE	Treatment	5	R3	T3	0.5094	0.1774	0.3321	0.2811	36.74
HTE	Treatment	5.5	R1	T1	0.5143	0.2028	0.3115	0.2605	34.06
HTE	Treatment	5.5	R1	T2	0.5198	0.2008	0.3190	0.2680	35.03

Product	Condition	Concentration	Bio Rep	Technical Rep	Raw A570	Raw A600	A570−A600	Blank-corrected absorbance	Cell viability (%)
HTE	Treatment	5.5	R1	T3	0.5095	0.1946	0.3149	0.2639	34.49
HTE	Treatment	5.5	R2	T1	0.5907	0.1793	0.4114	0.3604	47.11
HTE	Treatment	5.5	R2	T2	0.5883	0.1746	0.4136	0.3626	47.40
HTE	Treatment	5.5	R2	T3	0.5828	0.1801	0.4027	0.3517	45.97
HTE	Treatment	5.5	R3	T1	0.4920	0.1862	0.3058	0.2548	33.30
HTE	Treatment	5.5	R3	T2	0.4951	0.1922	0.3029	0.2519	32.93
HTE	Treatment	5.5	R3	T3	0.4952	0.1861	0.3091	0.2581	33.74

Table S20. Blank absorbance values used for correction of the viability assay.

Product	Blank replicate	Raw A570	Raw A600	ΔA blank
CSE	B1	0.1588	0.1121	0.0467
CSE	B2	0.1669	0.1173	0.0495
CSE	B3	0.1633	0.1155	0.0478
HTE	B1	0.1752	0.1235	0.0517
HTE	B2	0.1701	0.1180	0.0521
HTE	B3	0.1707	0.1215	0.0492

Table S21. Intracellular ROS, well-level data (DHE, 90 min). Fold change is calculated from the blank-corrected values against the blank-corrected mean vehicle (0-puff) value of the same biological replicate.

Product	Condition	Concentration	Bio Rep	Technical Rep	Raw DHE RFU	Blank-corrected RFU	Mean blank-corrected 0-puff RFU (same Bio Rep)	ROS Fold
CSE	C	N/A	R1	T1	9464.3	9428.9	9140.0	1.0316
CSE	C	N/A	R1	T2	9382.5	9347.1	9140.0	1.0227
CSE	C	N/A	R1	T3	9315.3	9279.9	9140.0	1.0153
CSE	C	N/A	R2	T1	8537.3	8503.4	8594.8	0.9894
CSE	C	N/A	R2	T2	8455.5	8421.6	8594.8	0.9798
CSE	C	N/A	R2	T3	8654.6	8620.7	8594.8	1.0030
CSE	C	N/A	R3	T1	9639.1	9605.2	9417.3	1.0200
CSE	C	N/A	R3	T2	9548.4	9514.5	9417.3	1.0103
CSE	C	N/A	R3	T3	9492.1	9458.2	9417.3	1.0043
CSE	0 puff	0	R1	T1	9241.3	9205.9	9140.0	1.0072
CSE	0 puff	0	R1	T2	9167.1	9131.7	9140.0	0.9991
CSE	0 puff	0	R1	T3	9117.8	9082.4	9140.0	0.9937
CSE	0 puff	0	R2	T1	8613.6	8579.7	8594.8	0.9982
CSE	0 puff	0	R2	T2	8535.8	8501.9	8594.8	0.9892
CSE	0 puff	0	R2	T3	8736.7	8702.8	8594.8	1.0126
CSE	0 puff	0	R3	T1	9451.3	9417.4	9417.3	1.0000
CSE	0 puff	0	R3	T2	9542.7	9508.8	9417.3	1.0097
CSE	0 puff	0	R3	T3	9359.6	9325.7	9417.3	0.9903
CSE	Treatment	0.1	R1	T1	13459.6	13424.2	9140.0	1.4687
CSE	Treatment	0.1	R1	T2	13545.7	13510.3	9140.0	1.4782

Product	Condition	Concentration	Bio Rep	Technical Rep	Raw DHE RFU	Blank-corrected RFU	Mean blank-corrected 0-puff RFU (same Bio Rep)	ROS Fold
CSE	Treatment	0.1	R1	T3	13270.5	13235.1	9140.0	1.4480
CSE	Treatment	0.1	R2	T1	12907.4	12873.5	8594.8	1.4978
CSE	Treatment	0.1	R2	T2	13058.2	13024.3	8594.8	1.5154
CSE	Treatment	0.1	R2	T3	12778.4	12744.5	8594.8	1.4828
CSE	Treatment	0.1	R3	T1	13880.1	13846.2	9417.3	1.4703
CSE	Treatment	0.1	R3	T2	13654.8	13620.9	9417.3	1.4464
CSE	Treatment	0.1	R3	T3	13508.0	13474.1	9417.3	1.4308
CSE	Treatment	0.3	R1	T1	18200.5	18165.1	9140.0	1.9874
CSE	Treatment	0.3	R1	T2	18058.3	18022.9	9140.0	1.9719
CSE	Treatment	0.3	R1	T3	17981.3	17945.9	9140.0	1.9634
CSE	Treatment	0.3	R2	T1	17918.5	17884.6	8594.8	2.0809
CSE	Treatment	0.3	R2	T2	17574.3	17540.4	8594.8	2.0408
CSE	Treatment	0.3	R2	T3	17789.4	17755.5	8594.8	2.0658
CSE	Treatment	0.3	R3	T1	19869.8	19835.9	9417.3	2.1063
CSE	Treatment	0.3	R3	T2	19615.6	19581.7	9417.3	2.0793
CSE	Treatment	0.3	R3	T3	19726.8	19692.9	9417.3	2.0911
CSE	Treatment	0.5	R1	T1	25386.5	25351.1	9140.0	2.7736
CSE	Treatment	0.5	R1	T2	25204.1	25168.7	9140.0	2.7537
CSE	Treatment	0.5	R1	T3	25520.0	25484.6	9140.0	2.7882
CSE	Treatment	0.5	R2	T1	22755.9	22722.0	8594.8	2.6437
CSE	Treatment	0.5	R2	T2	23392.2	23358.3	8594.8	2.7177
CSE	Treatment	0.5	R2	T3	23431.0	23397.1	8594.8	2.7222
CSE	Treatment	0.5	R3	T1	25154.7	25120.8	9417.3	2.6675
CSE	Treatment	0.5	R3	T2	25081.7	25047.8	9417.3	2.6598
CSE	Treatment	0.5	R3	T3	25537.0	25503.1	9417.3	2.7081
CSE	Treatment	0.7	R1	T1	28786.8	28751.4	9140.0	3.1457
CSE	Treatment	0.7	R1	T2	28980.8	28945.4	9140.0	3.1669
CSE	Treatment	0.7	R1	T3	29299.9	29264.5	9140.0	3.2018
CSE	Treatment	0.7	R2	T1	27833.0	27799.1	8594.8	3.2344
CSE	Treatment	0.7	R2	T2	27304.1	27270.2	8594.8	3.1729
CSE	Treatment	0.7	R2	T3	27727.4	27693.5	8594.8	3.2221
CSE	Treatment	0.7	R3	T1	29120.9	29087.0	9417.3	3.0887
CSE	Treatment	0.7	R3	T2	29944.6	29910.7	9417.3	3.1761
CSE	Treatment	0.7	R3	T3	29285.9	29252.0	9417.3	3.1062
CSE	Treatment	1	R1	T1	32467.5	32432.1	9140.0	3.5484
CSE	Treatment	1	R1	T2	32970.1	32934.7	9140.0	3.6034
CSE	Treatment	1	R1	T3	33101.4	33066.0	9140.0	3.6177
CSE	Treatment	1	R2	T1	30215.5	30181.6	8594.8	3.5116
CSE	Treatment	1	R2	T2	30795.6	30761.7	8594.8	3.5791
CSE	Treatment	1	R2	T3	30182.1	30148.2	8594.8	3.5077
CSE	Treatment	1	R3	T1	32456.3	32422.4	9417.3	3.4429
CSE	Treatment	1	R3	T2	32856.1	32822.2	9417.3	3.4853

Product	Condition	Concentration	Bio Rep	Technical Rep	Raw DHE RFU	Blank-corrected RFU	Mean blank-corrected 0-puff RFU (same Bio Rep)	ROS Fold
CSE	Treatment	1	R3	T3	32086.7	32052.8	9417.3	3.4036
CSE	Antimycin A	AA 50 µM	R1	T1	40245.0	40209.6	9140.0	4.3993
CSE	Antimycin A	AA 50 µM	R1	T2	40800.1	40764.7	9140.0	4.4600
CSE	Antimycin A	AA 50 µM	R1	T3	41141.4	41106.0	9140.0	4.4974
CSE	Antimycin A	AA 50 µM	R2	T1	38236.8	38202.9	8594.8	4.4449
CSE	Antimycin A	AA 50 µM	R2	T2	38937.5	38903.6	8594.8	4.5264
CSE	Antimycin A	AA 50 µM	R2	T3	39015.7	38981.8	8594.8	4.5355
CSE	Antimycin A	AA 50 µM	R3	T1	43848.2	43814.3	9417.3	4.6525
CSE	Antimycin A	AA 50 µM	R3	T2	42519.4	42485.5	9417.3	4.5114
CSE	Antimycin A	AA 50 µM	R3	T3	43352.8	43318.9	9417.3	4.5999
HTE	C	N/A / Untreated	R1	T1	8982.0	8948.1	9004.7	0.9937
HTE	C	N/A / Untreated	R1	T2	8804.4	8770.5	9004.7	0.9740
HTE	C	N/A / Untreated	R1	T3	9013.1	8979.2	9004.7	0.9972
HTE	C	N/A / Untreated	R2	T1	9704.1	9670.2	9480.9	1.0200
HTE	C	N/A / Untreated	R2	T2	9639.1	9605.2	9480.9	1.0131
HTE	C	N/A / Untreated	R2	T3	9798.4	9764.5	9480.9	1.0299
HTE	C	N/A / Untreated	R3	T1	8828.6	8794.5	8755.4	1.0045
HTE	C	N/A / Untreated	R3	T2	8766.7	8732.6	8755.4	0.9974
HTE	C	N/A / Untreated	R3	T3	8715.3	8681.2	8755.4	0.9915
HTE	0 puff	0	R1	T1	8949.3	8915.4	9004.7	0.9901
HTE	0 puff	0	R1	T2	9038.7	9004.8	9004.7	1.0000
HTE	0 puff	0	R1	T3	9127.8	9093.9	9004.7	1.0099
HTE	0 puff	0	R2	T1	9437.4	9403.5	9480.9	0.9918
HTE	0 puff	0	R2	T2	9600.9	9567.0	9480.9	1.0091
HTE	0 puff	0	R2	T3	9506.2	9472.3	9480.9	0.9991
HTE	0 puff	0	R3	T1	8769.6	8735.5	8755.4	0.9977
HTE	0 puff	0	R3	T2	8871.6	8837.5	8755.4	1.0094
HTE	0 puff	0	R3	T3	8727.4	8693.3	8755.4	0.9929
HTE	Treatment	3	R1	T1	15757.1	15723.2	9004.7	1.7461
HTE	Treatment	3	R1	T2	16098.5	16064.6	9004.7	1.7840
HTE	Treatment	3	R1	T3	16091.8	16057.9	9004.7	1.7833
HTE	Treatment	3	R2	T1	17426.2	17392.3	9480.9	1.8345
HTE	Treatment	3	R2	T2	17220.4	17186.5	9480.9	1.8127
HTE	Treatment	3	R2	T3	17067.0	17033.1	9480.9	1.7966
HTE	Treatment	3	R3	T1	15009.1	14975.0	8755.4	1.7104
HTE	Treatment	3	R3	T2	15264.2	15230.1	8755.4	1.7395
HTE	Treatment	3	R3	T3	15222.5	15188.4	8755.4	1.7347
HTE	Treatment	4	R1	T1	18144.5	18110.6	9004.7	2.0112
HTE	Treatment	4	R1	T2	17844.8	17810.9	9004.7	1.9780
HTE	Treatment	4	R1	T3	17671.0	17637.1	9004.7	1.9587
HTE	Treatment	4	R2	T1	19178.4	19144.5	9480.9	2.0193
HTE	Treatment	4	R2	T2	19611.9	19578.0	9480.9	2.0650

Product	Condition	Concentration	Bio Rep	Technical Rep	Raw DHE RFU	Blank-corrected RFU	Mean blank-corrected 0-puff RFU (same Bio Rep)	ROS Fold
HTE	Treatment	4	R2	T3	19217.6	19183.7	9480.9	2.0234
HTE	Treatment	4	R3	T1	17298.9	17264.8	8755.4	1.9719
HTE	Treatment	4	R3	T2	17169.2	17135.1	8755.4	1.9571
HTE	Treatment	4	R3	T3	17432.5	17398.4	8755.4	1.9872
HTE	Treatment	4.75	R1	T1	20413.3	20379.4	9004.7	2.2632
HTE	Treatment	4.75	R1	T2	20225.3	20191.4	9004.7	2.2423
HTE	Treatment	4.75	R1	T3	20073.6	20039.7	9004.7	2.2255
HTE	Treatment	4.75	R2	T1	21565.8	21531.9	9480.9	2.2711
HTE	Treatment	4.75	R2	T2	21914.8	21880.9	9480.9	2.3079
HTE	Treatment	4.75	R2	T3	22001.8	21967.9	9480.9	2.3171
HTE	Treatment	4.75	R3	T1	21109.7	21075.6	8755.4	2.4071
HTE	Treatment	4.75	R3	T2	20831.4	20797.3	8755.4	2.3754
HTE	Treatment	4.75	R3	T3	21099.3	21065.2	8755.4	2.4060
HTE	Treatment	5	R1	T1	23356.9	23323.0	9004.7	2.5901
HTE	Treatment	5	R1	T2	23284.3	23250.4	9004.7	2.5820
HTE	Treatment	5	R1	T3	23720.3	23686.4	9004.7	2.6304
HTE	Treatment	5	R2	T1	23847.5	23813.6	9480.9	2.5117
HTE	Treatment	5	R2	T2	23295.6	23261.7	9480.9	2.4535
HTE	Treatment	5	R2	T3	24043.3	24009.4	9480.9	2.5324
HTE	Treatment	5	R3	T1	22250.6	22216.5	8755.4	2.5375
HTE	Treatment	5	R3	T2	22257.5	22223.4	8755.4	2.5382
HTE	Treatment	5	R3	T3	21711.3	21677.2	8755.4	2.4759
HTE	Treatment	5.5	R1	T1	25328.2	25294.3	9004.7	2.8090
HTE	Treatment	5.5	R1	T2	24955.5	24921.6	9004.7	2.7676
HTE	Treatment	5.5	R1	T3	25356.6	25322.7	9004.7	2.8122
HTE	Treatment	5.5	R2	T1	26592.8	26558.9	9480.9	2.8013
HTE	Treatment	5.5	R2	T2	26179.8	26145.9	9480.9	2.7577
HTE	Treatment	5.5	R2	T3	26635.2	26601.3	9480.9	2.8058
HTE	Treatment	5.5	R3	T1	23225.0	23190.9	8755.4	2.6487
HTE	Treatment	5.5	R3	T2	22531.5	22497.4	8755.4	2.5695
HTE	Treatment	5.5	R3	T3	22743.0	22708.9	8755.4	2.5937
HTE	Antimycin A	AA 50 µM	R1	T1	41467.4	41433.5	9004.7	4.6013
HTE	Antimycin A	AA 50 µM	R1	T2	42397.8	42363.9	9004.7	4.7046
HTE	Antimycin A	AA 50 µM	R1	T3	41659.0	41625.1	9004.7	4.6226
HTE	Antimycin A	AA 50 µM	R2	T1	43861.8	43827.9	9480.9	4.6227
HTE	Antimycin A	AA 50 µM	R2	T2	44193.7	44159.8	9480.9	4.6577
HTE	Antimycin A	AA 50 µM	R2	T3	43244.3	43210.4	9480.9	4.5576
HTE	Antimycin A	AA 50 µM	R3	T1	38328.9	38294.8	8755.4	4.3738
HTE	Antimycin A	AA 50 µM	R3	T2	39372.4	39338.3	8755.4	4.4930
HTE	Antimycin A	AA 50 µM	R3	T3	39470.7	39436.6	8755.4	4.5042

Table S22. Per-replicate normalization values (mean blank-corrected vehicle RFU) and reagent-blank fluorescence for the DHE assay. Reagent blanks contained medium/buffer and DHE without cells or extract. Blank fluorescence was approximately 0.4% of the vehicle signal and was subtracted from every DHE reading before analysis; all DHE values reported in this article are blank-corrected.

Product	Bio Rep	Mean Normalization RFU	Reagent blank RFU (no cells)
CSE	R1	9175.4	35.4
CSE	R2	8628.7	33.9
CSE	R3	9451.2	33.9
HTE	R1	9038.6	33.9
HTE	R2	9514.8	33.9
HTE	R3	8789.5	34.1

Table S23. TNF-α In-Cell Western, well-level data (4 h).

Product	Concentration	Biological replicate	Technical replicate	Raw 800 nm	BG800 nm	Corrected 800 nm	Raw 700 nm	Normalized TNF-α
Shared	Control	R1	T1	18787.28	2126.35	16660.93	19989.99	0.8335
Shared	Control	R1	T2	18771.41	2064.76	16706.66	20391.52	0.8193
Shared	Control	R1	T3	18857.41	2022.92	16834.48	20417.00	0.8245
Shared	Control	R2	T1	19745.62	2184.87	17560.75	20896.93	0.8404
Shared	Control	R2	T2	19547.20	2174.72	17372.48	20816.88	0.8345
Shared	Control	R2	T3	20492.71	2326.67	18166.04	21312.16	0.8524
Shared	Control	R3	T1	19395.18	2184.99	17210.19	21652.51	0.7948
Shared	Control	R3	T2	18980.73	2066.20	16914.53	20984.02	0.8061
Shared	Control	R3	T3	19393.65	2110.46	17283.19	21195.99	0.8154
Shared	0	R1	T1	19759.32	2436.37	17322.95	21048.86	0.8230
Shared	0	R1	T2	19927.23	2360.16	17567.07	21374.26	0.8219
Shared	0	R1	T3	19231.55	2417.20	16814.35	20714.83	0.8117
Shared	0	R2	T1	19363.39	2318.03	17045.36	21520.95	0.7920
Shared	0	R2	T2	19180.83	2218.75	16962.08	21071.40	0.8050
Shared	0	R2	T3	19870.94	2273.88	17597.06	21584.08	0.8153
Shared	0	R3	T1	19936.41	2283.30	17653.12	21265.28	0.8301
Shared	0	R3	T2	20222.25	2234.15	17988.09	21124.37	0.8515
Shared	0	R3	T3	20154.76	2266.80	17887.96	21474.92	0.8330
CSE	0.1	R1	T1	24974.28	2516.61	22457.67	21239.05	1.0574
CSE	0.1	R1	T2	25014.82	2633.70	22381.12	20713.58	1.0805
CSE	0.1	R1	T3	25480.80	2531.98	22948.82	21168.96	1.0841
CSE	0.1	R2	T1	26008.36	2592.29	23416.07	21445.00	1.0919
CSE	0.1	R2	T2	25155.36	2520.32	22635.04	21044.78	1.0756
CSE	0.1	R2	T3	24834.95	2547.84	22287.11	20844.51	1.0692
CSE	0.1	R3	T1	24686.40	2418.42	22267.98	21531.21	1.0342
CSE	0.1	R3	T2	24516.50	2582.24	21934.26	21138.37	1.0377
CSE	0.1	R3	T3	24863.99	2501.69	22362.30	21222.45	1.0537
CSE	0.3	R1	T1	27956.10	3038.80	24917.30	18926.03	1.3166
CSE	0.3	R1	T2	27780.24	3050.98	24729.26	18707.29	1.3219

Product	Concentration	Biological replicate	Technical replicate	Raw 800 nm	BG800 nm	Corrected 800 nm	Raw 700 nm	Normalized TNF-α
CSE	0.3	R1	T3	28105.33	2890.25	25215.09	19323.76	1.3049
CSE	0.3	R2	T1	26336.03	2869.67	23466.36	18261.62	1.2850
CSE	0.3	R2	T2	25775.14	2696.80	23078.34	17864.22	1.2919
CSE	0.3	R2	T3	24920.97	2572.56	22348.41	17735.11	1.2601
CSE	0.3	R3	T1	26474.04	3120.31	23353.72	18447.46	1.2660
CSE	0.3	R3	T2	26537.34	3004.79	23532.55	18243.43	1.2899
CSE	0.3	R3	T3	26721.92	2984.80	23737.12	18500.31	1.2831
CSE	0.5	R1	T1	30324.67	3284.45	27040.21	16877.68	1.6021
CSE	0.5	R1	T2	31045.96	3533.08	27512.89	17256.74	1.5943
CSE	0.5	R1	T3	31194.08	3343.30	27850.78	17125.25	1.6263
CSE	0.5	R2	T1	33531.54	3444.27	30087.27	18264.11	1.6473
CSE	0.5	R2	T2	33049.40	3380.26	29669.14	18332.08	1.6184
CSE	0.5	R2	T3	33582.85	3569.56	30013.29	18065.40	1.6614
CSE	0.5	R3	T1	33405.52	3625.98	29779.54	18508.67	1.6090
CSE	0.5	R3	T2	32935.28	3364.64	29570.64	17999.78	1.6428
CSE	0.5	R3	T3	32497.97	3358.13	29139.83	17991.56	1.6196
CSE	0.7	R1	T1	33940.70	3559.61	30381.09	16337.04	1.8596
CSE	0.7	R1	T2	35475.33	3709.98	31765.35	16658.40	1.9069
CSE	0.7	R1	T3	34776.60	3728.02	31048.58	16574.25	1.8733
CSE	0.7	R2	T1	33601.95	3491.14	30110.82	16241.18	1.8540
CSE	0.7	R2	T2	33043.64	3419.29	29624.34	15970.40	1.8550
CSE	0.7	R2	T3	33395.57	3577.67	29817.90	16317.01	1.8274
CSE	0.7	R3	T1	37111.73	4048.87	33062.87	17194.53	1.9229
CSE	0.7	R3	T2	36361.21	3919.29	32441.92	16874.74	1.9225
CSE	0.7	R3	T3	37495.47	3950.08	33545.39	17023.83	1.9705
CSE	1.0	R1	T1	36076.89	3457.83	32619.06	15469.36	2.1086
CSE	1.0	R1	T2	36313.95	3464.98	32848.97	15354.20	2.1394
CSE	1.0	R1	T3	35952.56	3256.74	32695.82	15152.42	2.1578
CSE	1.0	R2	T1	34429.37	3526.85	30902.52	14215.83	2.1738
CSE	1.0	R2	T2	33664.73	3483.56	30181.17	14060.21	2.1466
CSE	1.0	R2	T3	33382.09	3241.74	30140.35	13837.28	2.1782
CSE	1.0	R3	T1	35900.88	3497.73	32403.15	15096.71	2.1464
CSE	1.0	R3	T2	35747.92	3511.97	32235.94	14824.01	2.1746
CSE	1.0	R3	T3	36183.29	3696.85	32486.44	15049.84	2.1586
HTE	3.0	R1	T1	23146.56	2362.97	20783.58	22220.25	0.9353
HTE	3.0	R1	T2	22859.97	2244.63	20615.33	22514.10	0.9157
HTE	3.0	R1	T3	23325.36	2387.86	20937.50	22607.29	0.9261
HTE	3.0	R2	T1	23249.35	2406.19	20843.16	21706.38	0.9602
HTE	3.0	R2	T2	23546.87	2362.03	21184.84	21779.22	0.9727
HTE	3.0	R2	T3	22818.06	2331.50	20486.56	21270.18	0.9632
HTE	3.0	R3	T1	20412.47	1927.42	18485.05	18678.84	0.9896
HTE	3.0	R3	T2	20537.81	1871.16	18666.64	19297.66	0.9673

Product	Concentration	Biological replicate	Technical replicate	Raw 800 nm	BG800 nm	Corrected 800 nm	Raw 700 nm	Normalized TNF-α
HTE	3.0	R3	T3	20239.54	1982.27	18257.26	18855.76	0.9683
HTE	4.0	R1	T1	28645.29	3198.22	25447.08	20529.74	1.2395
HTE	4.0	R1	T2	28837.14	3387.73	25449.40	20719.34	1.2283
HTE	4.0	R1	T3	28369.07	3266.30	25102.77	20195.19	1.2430
HTE	4.0	R2	T1	24567.28	2690.12	21877.16	18591.39	1.1767
HTE	4.0	R2	T2	24243.77	2796.47	21447.29	18120.93	1.1836
HTE	4.0	R2	T3	24799.67	2743.47	22056.20	18504.06	1.1920
HTE	4.0	R3	T1	27035.78	3085.53	23950.25	20117.64	1.1905
HTE	4.0	R3	T2	26728.44	3100.28	23628.16	19787.12	1.1941
HTE	4.0	R3	T3	27828.39	3085.63	24742.76	20355.01	1.2156
HTE	4.75	R1	T1	30876.03	3312.53	27563.49	19202.11	1.4354
HTE	4.75	R1	T2	31449.41	3470.02	27979.39	18998.71	1.4727
HTE	4.75	R1	T3	30888.50	3282.50	27606.01	18836.95	1.4655
HTE	4.75	R2	T1	31216.09	3619.75	27596.34	18760.88	1.4710
HTE	4.75	R2	T2	30679.98	3344.43	27335.54	18360.76	1.4888
HTE	4.75	R2	T3	30553.29	3424.58	27128.71	18742.04	1.4475
HTE	4.75	R3	T1	29161.18	3275.58	25885.60	18208.70	1.4216
HTE	4.75	R3	T2	28972.27	3065.28	25907.00	18141.55	1.4280
HTE	4.75	R3	T3	29064.20	3221.82	25842.38	17768.90	1.4544
HTE	5.0	R1	T1	35226.92	3547.40	31679.52	19206.33	1.6494
HTE	5.0	R1	T2	35065.91	3417.89	31648.02	19241.71	1.6448
HTE	5.0	R1	T3	34756.36	3371.96	31384.40	18814.56	1.6681
HTE	5.0	R2	T1	34519.07	3652.16	30866.91	17403.54	1.7736
HTE	5.0	R2	T2	33915.21	3474.77	30440.44	17494.56	1.7400
HTE	5.0	R2	T3	33931.83	3656.56	30275.26	17097.25	1.7708
HTE	5.0	R3	T1	32700.99	3364.10	29336.90	17769.66	1.6510
HTE	5.0	R3	T2	33190.03	3608.49	29581.54	17993.56	1.6440
HTE	5.0	R3	T3	32751.16	3335.83	29415.33	17669.63	1.6647
HTE	5.5	R1	T1	36533.73	3643.99	32889.74	17460.24	1.8837
HTE	5.5	R1	T2	35178.61	3414.54	31764.07	16984.97	1.8701
HTE	5.5	R1	T3	35564.66	3691.43	31873.23	17260.84	1.8466
HTE	5.5	R2	T1	38260.98	3659.42	34601.55	17770.81	1.9471
HTE	5.5	R2	T2	37318.30	3527.08	33791.22	17377.70	1.9445
HTE	5.5	R2	T3	37449.58	3635.69	33813.88	17684.35	1.9121
HTE	5.5	R3	T1	36229.84	3614.90	32614.94	16800.16	1.9413
HTE	5.5	R3	T2	38152.25	3653.77	34498.48	17310.35	1.9929
HTE	5.5	R3	T3	37516.56	3765.64	33750.91	17103.25	1.9734

Table S24. IL-6 In-Cell Western, well-level data (4 h).

Product	Concentration	Biological replicate	Technical replicate	Raw 800 nm	BG800 nm	Corrected 800 nm	Raw 700 nm	Normalized IL-6
Shared	Control	R1	T1	9980.21	1106.31	8873.90	21066.70	0.4212
Shared	Control	R1	T2	9869.29	1084.24	8785.05	20744.45	0.4235
Shared	Control	R1	T3	10083.63	1175.37	8908.26	21366.26	0.4169
Shared	Control	R2	T1	9101.99	1029.28	8072.71	20407.64	0.3956
Shared	Control	R2	T2	8986.26	965.39	8020.87	19996.30	0.4011
Shared	Control	R2	T3	8977.00	979.46	7997.54	20033.07	0.3992
Shared	Control	R3	T1	9549.06	1088.64	8460.43	21246.74	0.3982
Shared	Control	R3	T2	9419.91	1024.09	8395.82	21153.56	0.3969
Shared	Control	R3	T3	9196.49	985.97	8210.52	20905.73	0.3927
Shared	0	R1	T1	10438.41	1170.65	9267.75	21715.61	0.4268
Shared	0	R1	T2	10899.99	1226.98	9673.00	22234.87	0.4350
Shared	0	R1	T3	10560.26	1107.10	9453.16	21955.46	0.4306
Shared	0	R2	T1	10520.90	1204.88	9316.03	22559.16	0.4130
Shared	0	R2	T2	10402.83	1223.73	9179.10	22247.40	0.4126
Shared	0	R2	T3	10146.86	1226.88	8919.99	21897.94	0.4073
Shared	0	R3	T1	10102.23	1146.02	8956.21	20048.85	0.4467
Shared	0	R3	T2	10282.05	1184.52	9097.53	20703.64	0.4394
Shared	0	R3	T3	10340.25	1256.92	9083.33	20279.68	0.4479
CSE	0.1	R1	T1	12810.39	1424.00	11386.39	20018.59	0.5688
CSE	0.1	R1	T2	12724.10	1411.82	11312.28	19624.11	0.5764
CSE	0.1	R1	T3	12892.29	1516.05	11376.24	19848.60	0.5732
CSE	0.1	R2	T1	12872.53	1530.05	11342.48	19561.70	0.5798
CSE	0.1	R2	T2	12679.83	1398.98	11280.85	19144.79	0.5892
CSE	0.1	R2	T3	12426.20	1398.75	11027.45	18987.55	0.5808
CSE	0.1	R3	T1	12804.15	1496.43	11307.72	20860.80	0.5421
CSE	0.1	R3	T2	12708.02	1507.13	11200.89	21066.93	0.5317
CSE	0.1	R3	T3	12455.94	1524.73	10931.21	20647.69	0.5294
CSE	0.3	R1	T1	17264.00	1716.83	15547.17	20329.02	0.7648
CSE	0.3	R1	T2	16924.76	1702.61	15222.16	19862.47	0.7664
CSE	0.3	R1	T3	16558.01	1563.83	14994.19	20023.00	0.7488
CSE	0.3	R2	T1	18243.14	1864.78	16378.35	19490.50	0.8403
CSE	0.3	R2	T2	17839.04	1885.84	15953.20	19232.62	0.8295
CSE	0.3	R2	T3	17772.87	1803.96	15968.91	18868.15	0.8463
CSE	0.3	R3	T1	18562.37	1722.78	16839.58	19012.70	0.8857
CSE	0.3	R3	T2	18922.59	1896.28	17026.31	19023.99	0.8950
CSE	0.3	R3	T3	18779.71	1803.34	16976.37	18622.36	0.9116
CSE	0.5	R1	T1	20191.85	2250.26	17941.59	17604.64	1.0191
CSE	0.5	R1	T2	19933.75	2148.03	17785.71	17358.06	1.0246
CSE	0.5	R1	T3	20411.97	2183.16	18228.82	17409.72	1.0470

Product	Concentration	Biological replicate	Technical replicate	Raw 800 nm	BG800 nm	Corrected 800 nm	Raw 700 nm	Normalized IL-6
CSE	0.5	R2	T1	21901.45	2390.36	19511.10	17994.89	1.0843
CSE	0.5	R2	T2	21335.35	2293.29	19042.05	17866.72	1.0658
CSE	0.5	R2	T3	21840.84	2499.86	19340.98	18118.82	1.0675
CSE	0.5	R3	T1	19634.32	2100.40	17533.92	17534.23	1.0000
CSE	0.5	R3	T2	19816.29	2024.74	17791.55	17744.41	1.0027
CSE	0.5	R3	T3	19612.80	2041.43	17571.37	17340.42	1.0133
CSE	0.7	R1	T1	25893.63	2675.90	23217.72	16959.92	1.3690
CSE	0.7	R1	T2	26160.18	2603.19	23556.98	17037.60	1.3826
CSE	0.7	R1	T3	25928.96	2732.95	23196.01	16555.88	1.4011
CSE	0.7	R2	T1	23775.47	2577.67	21197.80	16666.69	1.2719
CSE	0.7	R2	T2	23339.19	2394.14	20945.05	16598.30	1.2619
CSE	0.7	R2	T3	23368.63	2365.11	21003.52	16422.20	1.2790
CSE	0.7	R3	T1	23510.43	2521.19	20989.24	16199.68	1.2957
CSE	0.7	R3	T2	23201.61	2448.53	20753.08	16111.97	1.2881
CSE	0.7	R3	T3	22986.09	2336.93	20649.16	16378.23	1.2608
CSE	1.0	R1	T1	28632.88	3232.29	25400.59	16867.70	1.5059
CSE	1.0	R1	T2	27901.91	3290.78	24611.13	16642.68	1.4788
CSE	1.0	R1	T3	27282.96	3014.70	24268.26	16367.18	1.4827
CSE	1.0	R2	T1	22723.47	2527.76	20195.71	15310.30	1.3191
CSE	1.0	R2	T2	23425.61	2512.89	20912.72	15575.06	1.3427
CSE	1.0	R2	T3	23693.34	2619.10	21074.24	15514.14	1.3584
CSE	1.0	R3	T1	24528.08	2983.49	21544.59	16189.11	1.3308
CSE	1.0	R3	T2	25673.86	2955.53	22718.32	16707.62	1.3598
CSE	1.0	R3	T3	25352.38	2877.32	22475.06	16557.18	1.3574
HTE	3.0	R1	T1	10507.16	1106.17	9400.99	20377.55	0.4613
HTE	3.0	R1	T2	10472.89	1066.06	9406.83	20359.19	0.4620
HTE	3.0	R1	T3	10911.17	1162.37	9748.80	20661.21	0.4718
HTE	3.0	R2	T1	10327.60	1107.04	9220.56	20859.88	0.4420
HTE	3.0	R2	T2	10307.01	1040.38	9266.63	20514.61	0.4517
HTE	3.0	R2	T3	10094.49	1052.46	9042.03	20543.14	0.4401
HTE	3.0	R3	T1	10411.11	1068.19	9342.92	21852.68	0.4275
HTE	3.0	R3	T2	10371.57	1033.01	9338.55	21624.24	0.4319
HTE	3.0	R3	T3	10674.54	1100.06	9574.48	22117.95	0.4329
HTE	4.0	R1	T1	13915.14	1620.20	12294.93	20342.03	0.6044
HTE	4.0	R1	T2	13177.77	1477.97	11699.80	19728.69	0.5930
HTE	4.0	R1	T3	13407.59	1476.01	11931.58	19852.66	0.6010
HTE	4.0	R2	T1	13981.83	1549.65	12432.18	20390.13	0.6097
HTE	4.0	R2	T2	14245.52	1656.85	12588.66	20309.48	0.6198
HTE	4.0	R2	T3	14069.42	1567.92	12501.50	20675.37	0.6047
HTE	4.0	R3	T1	11616.69	1299.83	10316.86	18973.15	0.5438
HTE	4.0	R3	T2	11610.77	1351.80	10258.97	18711.77	0.5483
HTE	4.0	R3	T3	11808.87	1363.65	10445.22	18713.27	0.5582

Product	Concentration	Biological replicate	Technical replicate	Raw 800 nm	BG800 nm	Corrected 800 nm	Raw 700 nm	Normalized IL-6
HTE	4.75	R1	T1	17320.59	1906.93	15413.66	19441.73	0.7928
HTE	4.75	R1	T2	17510.12	1959.11	15551.01	19884.95	0.7820
HTE	4.75	R1	T3	16533.80	1715.99	14817.81	19240.27	0.7701
HTE	4.75	R2	T1	17451.95	1773.56	15678.39	18662.68	0.8401
HTE	4.75	R2	T2	18117.97	1976.07	16141.89	18856.10	0.8561
HTE	4.75	R2	T3	17962.70	1921.90	16040.80	18949.54	0.8465
HTE	4.75	R3	T1	17762.05	1917.25	15844.80	18634.02	0.8503
HTE	4.75	R3	T2	17940.32	1920.34	16019.98	18476.42	0.8671
HTE	4.75	R3	T3	17460.07	1797.61	15662.46	18088.17	0.8659
HTE	5.0	R1	T1	18711.45	1888.49	16822.96	17532.74	0.9595
HTE	5.0	R1	T2	18839.80	1786.85	17052.95	17551.01	0.9716
HTE	5.0	R1	T3	19017.56	1842.15	17175.41	17990.63	0.9547
HTE	5.0	R2	T1	20631.35	2071.15	18560.19	18845.64	0.9849
HTE	5.0	R2	T2	20281.45	1915.84	18365.61	18623.05	0.9862
HTE	5.0	R2	T3	19612.81	1985.56	17627.25	18333.25	0.9615
HTE	5.0	R3	T1	20922.05	1999.21	18922.83	18438.88	1.0262
HTE	5.0	R3	T2	21788.04	2251.79	19536.26	18768.00	1.0409
HTE	5.0	R3	T3	20516.17	2029.76	18486.41	18208.76	1.0152
HTE	5.5	R1	T1	23621.84	2563.58	21058.26	15743.11	1.3376
HTE	5.5	R1	T2	24532.49	2704.93	21827.56	15890.56	1.3736
HTE	5.5	R1	T3	24497.02	2545.22	21951.80	16211.58	1.3541
HTE	5.5	R2	T1	23211.31	2643.67	20567.64	17185.44	1.1968
HTE	5.5	R2	T2	24386.51	2682.67	21703.84	17706.01	1.2258
HTE	5.5	R2	T3	24390.97	2716.05	21674.92	17631.87	1.2293
HTE	5.5	R3	T1	22889.69	2604.40	20285.29	16408.79	1.2362
HTE	5.5	R3	T2	21766.00	2394.47	19371.53	15977.00	1.2125
HTE	5.5	R3	T3	22178.06	2465.32	19712.74	16239.09	1.2139

Table S25. ATG5 In-Cell Western, well-level data (4 h).

Product	Concentration	Biological replicate	Technical replicate	Raw 800 nm	Background 800 nm	Corrected 800 nm	Raw 700 nm	800/700 normalized ratio	Relative expression (%)
Shared	Control	R1	T1	16959.05	1599.73	15359.32	28581.15	0.537393	96.83
Shared	Control	R1	T2	16552.87	1491.40	15061.48	28479.50	0.528853	95.29
Shared	Control	R1	T3	16329.44	1466.55	14862.89	27754.88	0.535506	96.49
Shared	Control	R2	T1	14715.92	1331.44	13384.49	25809.69	0.518584	93.44
Shared	Control	R2	T2	14989.19	1403.91	13585.28	26588.87	0.510939	92.06
Shared	Control	R2	T3	15010.67	1403.30	13607.37	25811.61	0.527180	94.99
Shared	Control	R3	T1	19387.37	1828.17	17559.21	28574.23	0.614512	110.72
Shared	Control	R3	T2	19453.07	1808.76	17644.31	28641.35	0.616043	111.00
Shared	Control	R3	T3	18777.91	1676.05	17101.85	28221.37	0.605989	109.19
Shared	0	R1	T1	7533.01	736.90	6796.12	26752.71	0.254035	45.77
Shared	0	R1	T2	7448.58	716.56	6732.03	27124.49	0.248190	44.72

Product	Concentration	Biological replicate	Technical replicate	Raw 800 nm	Background 800 nm	Corrected 800 nm	Raw 700 nm	800/700 normalized ratio	Relative expression (%)
Shared	0	R1	T3	7683.60	723.14	6960.46	27336.36	0.254623	45.88
Shared	0	R2	T1	4575.48	453.94	4121.54	26491.11	0.155582	28.03
Shared	0	R2	T2	4397.27	442.97	3954.30	26340.45	0.150123	27.05
Shared	0	R2	T3	4441.90	425.56	4016.34	26011.04	0.154409	27.82
Shared	0	R3	T1	6021.25	556.33	5464.92	27622.34	0.197844	35.65
Shared	0	R3	T2	6076.05	584.78	5491.27	27100.90	0.202623	36.51
Shared	0	R3	T3	6014.99	587.98	5427.01	27846.09	0.194893	35.12
CSE	0.1	R1	T1	9072.25	927.41	8144.84	26147.95	0.311491	56.12
CSE	0.1	R1	T2	9369.74	985.79	8383.95	26396.77	0.317613	57.23
CSE	0.1	R1	T3	8816.38	894.43	7921.96	25534.32	0.310247	55.90
CSE	0.1	R2	T1	12570.26	1293.87	11276.40	28625.85	0.393924	70.98
CSE	0.1	R2	T2	12290.18	1210.39	11079.80	28180.34	0.393175	70.84
CSE	0.1	R2	T3	11846.93	1252.28	10594.65	27868.80	0.380162	68.50
CSE	0.1	R3	T1	13322.34	1294.62	12027.73	27180.00	0.442521	79.73
CSE	0.1	R3	T2	13264.60	1381.75	11882.85	27361.45	0.434292	78.25
CSE	0.1	R3	T3	13336.18	1342.27	11993.90	26882.42	0.446162	80.39
CSE	0.3	R1	T1	9987.42	995.87	8991.54	24545.93	0.366315	66.00
CSE	0.3	R1	T2	10335.00	969.87	9365.13	25116.80	0.372863	67.18
CSE	0.3	R1	T3	9910.89	968.39	8942.50	24337.31	0.367440	66.21
CSE	0.3	R2	T1	9545.48	949.07	8596.41	25080.69	0.342750	61.76
CSE	0.3	R2	T2	9407.55	940.97	8466.58	24803.24	0.341350	61.50
CSE	0.3	R2	T3	9015.94	870.23	8145.71	24438.80	0.333311	60.06
CSE	0.3	R3	T1	14143.80	1409.82	12733.98	25487.89	0.499609	90.02
CSE	0.3	R3	T2	13775.34	1396.05	12379.29	24793.85	0.499289	89.96
CSE	0.3	R3	T3	13939.43	1438.05	12501.38	25651.33	0.487358	87.81
CSE	0.5	R1	T1	12370.43	1156.74	11213.68	22970.77	0.488172	87.96
CSE	0.5	R1	T2	12293.96	1173.37	11120.59	22459.12	0.495148	89.22
CSE	0.5	R1	T3	12078.62	1082.78	10995.83	22840.96	0.481409	86.74
CSE	0.5	R2	T1	17702.43	1845.61	15856.82	21476.82	0.738323	133.03
CSE	0.5	R2	T2	17691.37	1723.94	15967.43	21788.36	0.732842	132.04
CSE	0.5	R2	T3	17510.48	1724.40	15786.07	21850.20	0.722468	130.17
CSE	0.5	R3	T1	9365.63	897.99	8467.63	22822.88	0.371015	66.85
CSE	0.5	R3	T2	9317.25	867.60	8449.65	23172.83	0.364636	65.70
CSE	0.5	R3	T3	9392.50	873.47	8519.02	23292.51	0.365741	65.90
CSE	0.7	R1	T1	14294.58	1510.49	12784.09	20170.43	0.633803	114.20
CSE	0.7	R1	T2	14378.55	1406.96	12971.59	19921.63	0.651131	117.32
CSE	0.7	R1	T3	14932.80	1519.64	13413.15	20520.20	0.653656	117.78
CSE	0.7	R2	T1	9996.95	1009.95	8987.00	18602.58	0.483105	87.05
CSE	0.7	R2	T2	10010.45	1067.90	8942.55	19116.16	0.467801	84.29
CSE	0.7	R2	T3	10015.79	990.37	9025.42	19165.85	0.470912	84.85
CSE	0.7	R3	T1	16602.53	1745.27	14857.26	18446.56	0.805422	145.12
CSE	0.7	R3	T2	17002.77	1674.82	15327.94	18911.47	0.810511	146.04

Product	Concentration	Biological replicate	Technical replicate	Raw 800 nm	Background 800 nm	Corrected 800 nm	Raw 700 nm	800/700 normalized ratio	Relative expression (%)
CSE	0.7	R3	T3	16574.42	1679.37	14895.05	18639.56	0.799109	143.98
CSE	1.0	R1	T1	18093.32	1819.31	16274.02	18019.82	0.903118	162.72
CSE	1.0	R1	T2	18524.20	1948.77	16575.42	17930.40	0.924431	166.56
CSE	1.0	R1	T3	18274.90	1889.77	16385.13	17762.21	0.922471	166.21
CSE	1.0	R2	T1	15881.51	1539.61	14341.90	17216.57	0.833029	150.10
CSE	1.0	R2	T2	16311.08	1669.64	14641.44	17497.39	0.836779	150.77
CSE	1.0	R2	T3	15913.73	1655.83	14257.90	17404.59	0.819204	147.60
CSE	1.0	R3	T1	9967.36	1038.91	8928.45	17520.71	0.509594	91.82
CSE	1.0	R3	T2	10521.05	1101.71	9419.34	18054.80	0.521709	94.00
CSE	1.0	R3	T3	10554.40	1178.84	9375.55	17904.30	0.523648	94.35
HTE	3.0	R1	T1	13700.78	1461.95	12238.83	25397.42	0.481893	86.83
HTE	3.0	R1	T2	12917.94	1362.32	11555.62	24909.95	0.463896	83.58
HTE	3.0	R1	T3	13425.65	1350.05	12075.60	25281.76	0.477641	86.06
HTE	3.0	R2	T1	17723.54	1772.24	15951.31	28662.32	0.556525	100.27
HTE	3.0	R2	T2	16927.53	1712.84	15214.68	27743.75	0.548400	98.81
HTE	3.0	R2	T3	17540.80	1813.56	15727.24	28382.02	0.554127	99.84
HTE	3.0	R3	T1	10408.27	1039.52	9368.75	26231.22	0.357160	64.35
HTE	3.0	R3	T2	10910.66	1139.33	9771.33	26649.76	0.366657	66.06
HTE	3.0	R3	T3	10813.59	1136.25	9677.34	26512.80	0.365006	65.77
HTE	4.0	R1	T1	14995.13	1526.57	13468.56	22983.52	0.586009	105.59
HTE	4.0	R1	T2	15451.36	1695.34	13756.02	23598.18	0.582927	105.03
HTE	4.0	R1	T3	15678.79	1641.41	14037.38	23297.78	0.602520	108.56
HTE	4.0	R2	T1	14796.25	1512.78	13283.47	25896.38	0.512947	92.42
HTE	4.0	R2	T2	15079.73	1549.99	13529.74	25545.77	0.529627	95.43
HTE	4.0	R2	T3	14656.61	1549.26	13107.36	25055.33	0.523136	94.26
HTE	4.0	R3	T1	10092.34	1030.31	9062.03	24539.57	0.369282	66.54
HTE	4.0	R3	T2	9839.99	1027.28	8812.71	24571.03	0.358663	64.62
HTE	4.0	R3	T3	9958.58	986.47	8972.10	25007.35	0.358779	64.64
HTE	4.75	R1	T1	11744.68	1236.56	10508.12	24502.32	0.428863	77.27
HTE	4.75	R1	T2	11819.70	1197.28	10622.42	24224.29	0.438503	79.01
HTE	4.75	R1	T3	11188.67	1126.17	10062.50	23727.14	0.424092	76.41
HTE	4.75	R2	T1	16722.06	1761.19	14960.88	22919.67	0.652753	117.61
HTE	4.75	R2	T2	16345.32	1707.61	14637.71	22780.02	0.642568	115.78
HTE	4.75	R2	T3	16618.10	1687.00	14931.10	23127.82	0.645590	116.32
HTE	4.75	R3	T1	11866.24	1204.81	10661.42	23089.59	0.461742	83.20
HTE	4.75	R3	T2	11402.60	1139.45	10263.16	22756.01	0.451009	81.26
HTE	4.75	R3	T3	11366.56	1089.29	10277.27	22679.36	0.453155	81.65
HTE	5.0	R1	T1	11868.92	1118.47	10750.45	21828.31	0.492501	88.74
HTE	5.0	R1	T2	11589.40	1048.68	10540.72	21204.96	0.497087	89.57
HTE	5.0	R1	T3	11956.23	1152.24	10803.99	21436.39	0.504002	90.81
HTE	5.0	R2	T1	13770.61	1286.37	12484.25	20117.08	0.620580	111.82
HTE	5.0	R2	T2	14322.01	1390.86	12931.15	20205.42	0.639984	115.31

Product	Concentration	Biological replicate	Technical replicate	Raw 800 nm	Background 800 nm	Corrected 800 nm	Raw 700 nm	800/700 normalized ratio	Relative expression (%)
HTE	5.0	R2	T3	13790.40	1350.96	12439.44	19732.49	0.630404	113.59
HTE	5.0	R3	T1	8794.54	812.38	7982.16	19251.32	0.414629	74.71
HTE	5.0	R3	T2	9207.29	862.86	8344.43	19930.44	0.418678	75.44
HTE	5.0	R3	T3	9160.45	892.06	8268.40	19530.25	0.423364	76.28
HTE	5.5	R1	T1	11508.65	1123.42	10385.23	18761.29	0.553546	99.74
HTE	5.5	R1	T2	11488.05	1094.89	10393.16	18175.08	0.571836	103.03
HTE	5.5	R1	T3	11168.03	1049.43	10118.60	18235.77	0.554876	99.98
HTE	5.5	R2	T1	11928.00	1156.97	10771.02	16611.48	0.648409	116.83
HTE	5.5	R2	T2	11956.58	1163.95	10792.63	16886.82	0.639116	115.16
HTE	5.5	R2	T3	12180.66	1235.44	10945.22	16701.44	0.655346	118.08
HTE	5.5	R3	T1	8782.10	869.95	7912.15	19015.50	0.416090	74.97
HTE	5.5	R3	T2	9029.56	836.20	8193.35	19460.01	0.421035	75.86
HTE	5.5	R3	T3	8960.91	873.05	8087.86	19449.63	0.415836	74.93
